# The macrophage migration inhibitory factor pathway in human B cells is tightly controlled and dysregulated in multiple sclerosis

**DOI:** 10.1002/eji.201847623

**Published:** 2018-09-25

**Authors:** Liza Rijvers, Marie‐José Melief, Roos M. van der Vuurst de Vries, Maeva Stéphant, Jamie van Langelaar, Annet F. Wierenga‐Wolf, Jeanet M. Hogervorst, Anneke J. Geurts‐Moespot, Fred C. G. J. Sweep, Rogier Q. Hintzen, Marvin M. van Luijn

**Affiliations:** ^1^ Department of Immunology Erasmus MC University Medical Center Rotterdam The Netherlands; ^2^ Department of Neurology Erasmus MC University Medical Center Rotterdam The Netherlands; ^3^ MS Center ErasMS Erasmus MC University Medical Center Rotterdam The Netherlands; ^4^ Department of Laboratory Medicine Radboud University Medical Center Nijmegen The Netherlands

**Keywords:** Autoimmune disease, B‐cell biology, Clinically isolated syndrome, MIF receptors CXCR4/CD74, MS

## Abstract

In MS, B cells survive peripheral tolerance checkpoints to mediate local inflammation, but the underlying molecular mechanisms are relatively underexplored. In mice, the MIF pathway controls B‐cell development and the induction of EAE. Here, we found that MIF and MIF receptor CD74 are downregulated, while MIF receptor CXCR4 is upregulated in B cells from early onset MS patients. B cells were identified as the main immune subset in blood expressing MIF. Blocking of MIF and CD74 signaling in B cells triggered CXCR4 expression, and vice versa, with separate effects on their proinflammatory activity, proliferation, and sensitivity to Fas‐mediated apoptosis. This study reveals a new reciprocal negative regulation loop between CD74 and CXCR4 in human B cells. The disturbance of this loop during MS onset provides further insights into how pathogenic B cells survive peripheral tolerance checkpoints to mediate disease activity in MS.

## Introduction

MS is a chronic autoimmune disease of the CNS, in which infiltrating proinflammatory immune cells mediate local pathology 1. The strong effects of anti‐CD20 monoclonal antibody therapy in MS patients demonstrate a key role for peripheral B cells during the pathogenesis 2. Immature B cells survive peripheral tolerance checkpoints in MS 3, but underlying mechanisms are poorly understood.

An important factor associated with chronic inflammation and cell survival is MIF 4. In the animal model of MS, i.e. EAE, MIF levels are increased in the CNS 5. Furthermore, anti‐MIF treatment prevents disease onset and improves the course of the disease by decreasing the expression of VCAM‐1, which impairs the homing of neuroantigen‐specific T cells to the CNS 6. Which and how immune subsets are regulated by MIF to promote disease activity in MS patients remains to be determined.

In murine B cells, triggering of the cognate receptor of MIF, CD74 (invariant chain), results in enhanced proliferation and proinflammatory cytokine production via NF‐κB 7, 8. Besides functioning as an MHC class II chaperone protein, CD74 also has an MHC class II‐independent role in B‐cell maturation 9. Interestingly, MIF is also a noncognate ligand for chemokine receptor CXCR4 10, which probably cooperate with CD74 to regulate B‐cell development and function through MIF 11, 12, 13.

This study explores whether MIF, CD74, and CXCR4 expression in B cells is associated with early MS disease activity, and how the regulation and downstream effects of MIF receptors CXCR4 and CD74 affect human B‐cell function. We show that MIF and CD74 are downregulated and CXCR4 is upregulated in blood B cells from early MS patients. This is dependent on a B cell‐intrinsic negative regulation loop between MIF, CXCR4, and CD74, which mediates their proinflammatory activity, proliferation, and sensitivity to Fas‐mediated apoptosis.

## Results

### The expression ratio of MIF receptors CXCR4 and CD74 on B cells is increased during rapid MS onset

**Figure 1:**
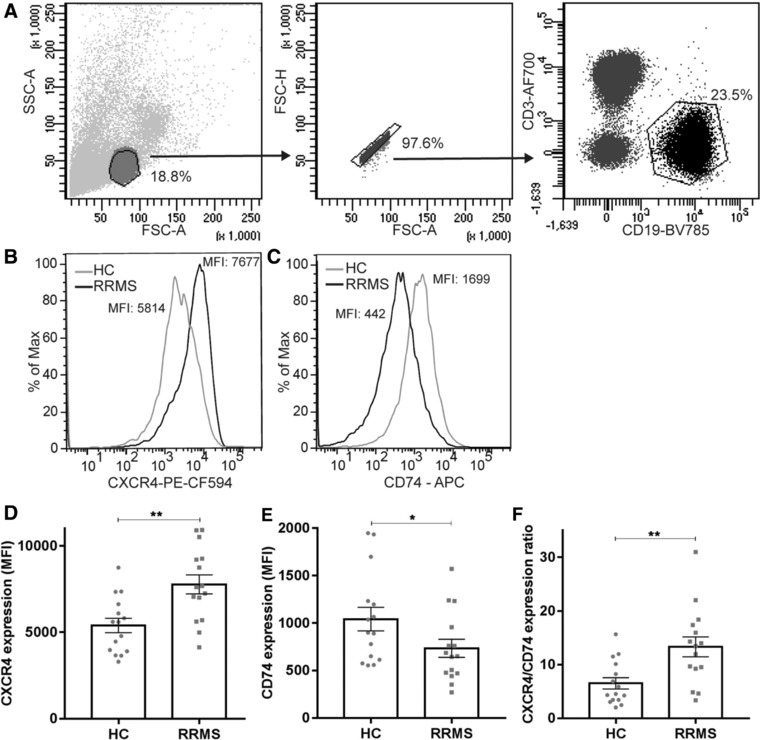
CXCR4 upregulation and CD74 downregulation on B cells of clinically definite MS patients. (A) Gating strategy for CD19+ B cells. (B and C) representative histograms of CXCR4 (B) and CD74 (C) expression levels in HC and RRMS. (D–F) Expression of MIF receptors CXCR4 (D) and CD74 (E) and their ratios (F) on blood B cells from 15 RRMS patients and 15 age‐ and gender‐matched healthy controls (HC) as determined by FACS. Data were measured in three individual experiments, with 5 HC and 5 RRMS patients per experiment. Data are shown as mean ± SEM. Unpaired *t*‐tests were used to compare groups. *p < 0.05, **p < 0.01.

To determine whether the B cell‐intrinsic MIF pathway is differentially regulated in early MS, we assessed the expression levels of MIF receptors CXCR4 and CD74 on blood B cells of relapsing‐remitting MS (RRMS) patients and healthy controls (HC). CXCR4 was 1.4‐fold increased on B cells from 15 RRMS patients compared to 15 age‐ and gender‐matched HC (*p* = 0.002, Fig. 1A, B, and D), which was reproduced and validated in additional cohorts (Supporting Information Fig. 1A and B). In contrast, CD74 expression was 1.4‐fold reduced on B cells in RRMS versus HC (*p* = 0.038, Fig. 1A, C, and E). The ratio of CXCR4 and CD74 expression levels on B cells was even further enhanced in RRMS (2.1‐fold, *p* = 0.004; Fig. 1F; Supporting Information Fig. 1C and D), suggesting that both MIF receptors are dysregulated on a per‐patient basis.

**Figure 2:**
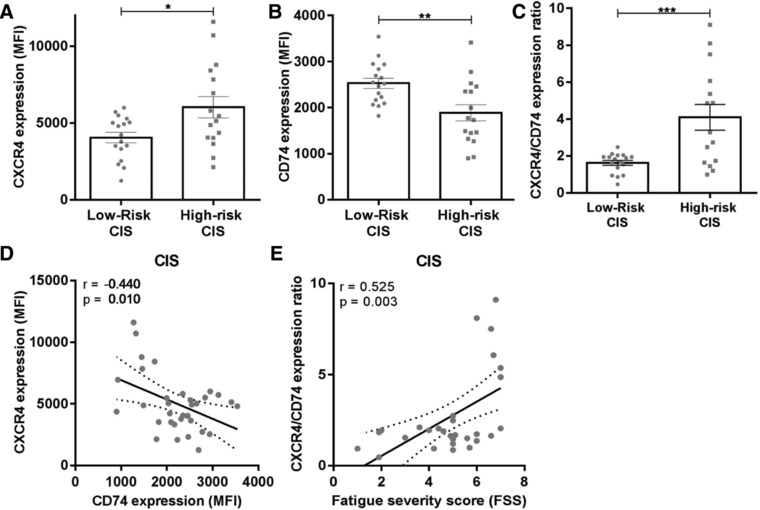
High CXCR4/CD74 expression ratios on B cells of CIS patients associate with rapid MS diagnosis. Expression of MIF receptors CXCR4 and CD74 and their ratios on blood B cells of 17 low‐risk CIS and 16 high‐risk CIS patients (A–C), as determined by FACS. Gating on CD19+ B cells is depicted in Fig. 1. Data were measured in nine individual experiments, with 1–2 low‐risk CIS and 1–2 high‐risk CIS patients were measured per experiment. Data are shown as mean ± SEM. Unpaired *t*‐tests were used to compare groups. (D) Correlation between CXCR4 and CD74 expression on B cells in CIS patients (*n* = 33). (E) Correlation between CXCR4/CD74 surface expression ratios on B cells and fatigue severity scores (FSS) for CIS patients (*n* = 30). *r* = Pearson's correlation coefficient (D) or Spearman correlation (E). *p < 0.05, **p < 0.01, ***p < 0.001.

Next to RRMS patients, patients with clinically isolated syndrome (CIS), a first manifestation of suspected MS 14, were analyzed. Similar to RRMS, B cells from CIS patients with a very rapid onset of clinically definite MS (CDMS) (“high‐risk CIS,” *n* = 16) showed 1.5‐fold increased CXCR4 (*p* = 0.014, Fig. 2A) and 1.3‐fold reduced CD74 surface levels (*p* = 0.004, Fig. 2B) compared to CIS patients with slow or no onset of CDMS (“low‐risk CIS,” *n* = 17). This resulted in strongly elevated CXCR4/CD74 expression ratios per patient in the high‐risk CIS group (2.5‐fold; Fig. 2C). In CIS, a negative correlation was found between CXCR4 and CD74 levels on B cells (*r* = –0.44, *P* = 0.01; Fig. 2D), and CXCR4/CD74 expression ratios positively associated with fatigue (*r* = 0.53, *P* = 0.003; Fig. 2E), an independent predictor of rapid CIS to CDMS transition 15.

**Figure 3:**
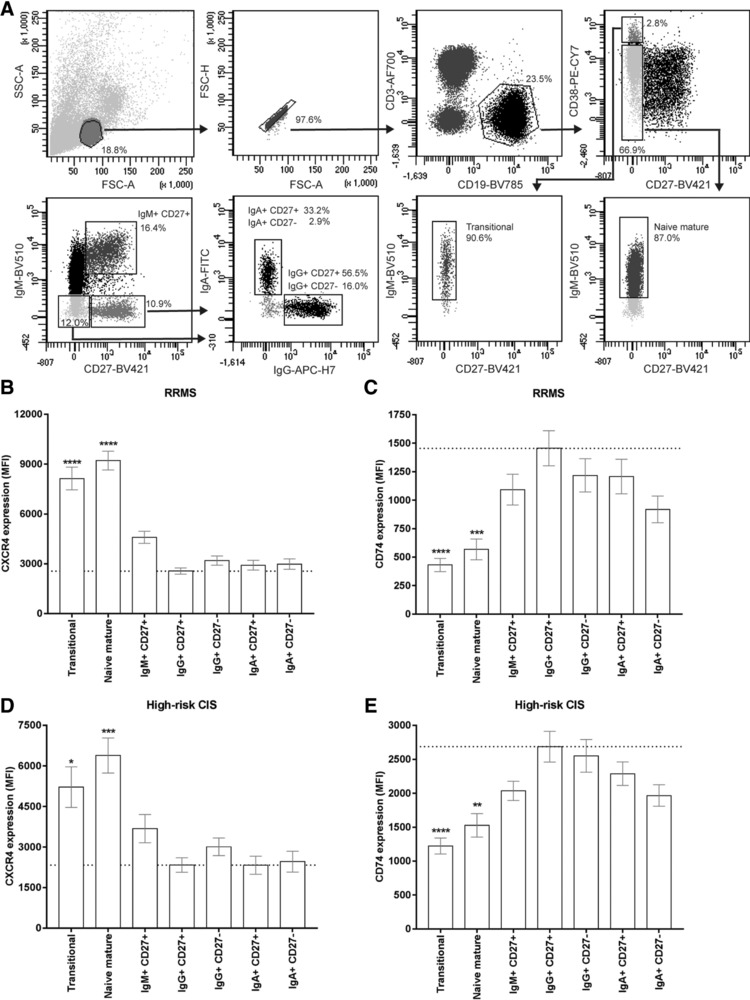
The CXCR4^hi^CD74^lo^ phenotype of B cells in early MS blood is linked to transitional and naive mature populations. (A) Used gating strategy for the different blood B‐cell subsets analyzed in B–E: transitional (IgM^+^CD27^−^CD38^hi^CD24^hi^), naive mature (IgM^+^CD27^−^CD38^−/dim^), IgM^+^CD27^+^ (nonclass‐switched memory), IgG^+^CD27^+^, IgG^+^CD27^−^, IgA^+^CD27^+^, and IgA^+^CD27^−^ B cells. Blood from RRMS (B, C; *n* = 15) and high‐risk CIS (D, E; *n* = 16) patients was used to assess CXCR4 (B, D) and CD74 (C, E) expression on these subsets. For RRMS patients, the data were measured in three individual experiments with five RRMS patients per experiment. For high‐risk CIS patients, data were measured in nine individual experiments with 1–2 patients per experiment. The expression level on each subset was compared to that of IgG^+^CD27^+^ memory B cells. Data are shown as mean ± SEM. Kruskal–Wallis test with Dunn's correction for multiple comparison was used to compare groups. *p < 0.05, **p < 0.01, ***p < 0.001, ****p < 0.0001.

In RRMS and high‐risk CIS blood, transitional (IgM^+^CD27^−^CD38^hi^CD24^hi^) as well as naive mature (IgM^+^CD27^−^CD38^−/dim^) B‐cell subsets displayed the highest CXCR4/CD74 expression ratios as compared to class‐switched (CD27^+^/CD27^−^ IgG^+^ and IgA^+^) and nonclass‐switched (IgM^+^CD27^+^) memory subsets (Fig. 3; Supporting Information Fig. 2), implying that the CXCR4^hi^CD74^lo^ phenotype of B cells in early MS reflects a more immature state. These data demonstrate that MIF receptors CXCR4 and CD74 are inversely expressed on B cells, which is dysregulated during early disease onset in MS.

### MIF is predominantly expressed by B cells in healthy blood and downregulated in early MS patients

**Figure 4:**
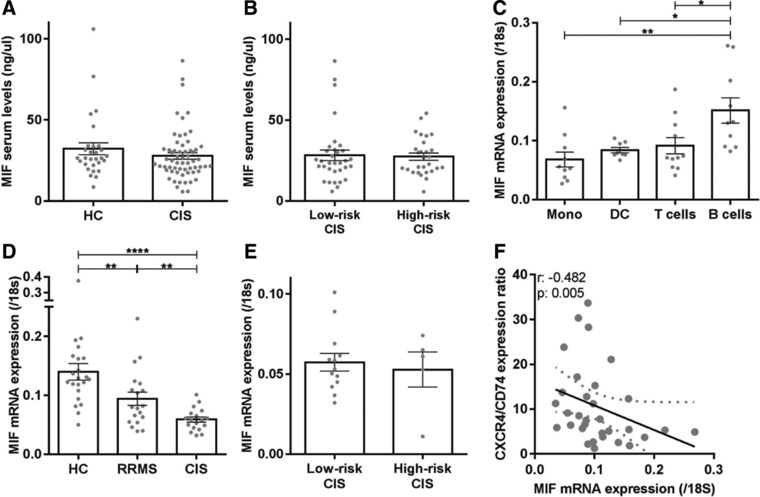
Serum MIF levels are not different, while the predominant MIF expression in B cells is reduced in CIS and RRMS patients. MIF serum levels (ng/μL) were compared between CIS patients (*n* = 61) and HC (*n* = 29; (A), as well as between low‐risk CIS (*n* = 33) and high‐risk CIS (*n* = 28) subgroups (B) using ELISA. Data are measured in one single experiment. Each dot represents the mean value of one individual, measured in duplicates. (C) Monocytes (*n* = 10), dendritic cells (*n* = 8), T cells (*n* = 11), and B cells (*n* = 10) were sorted from healthy blood and assessed for MIF mRNA expression relative to those of 18S mRNA using qPCR. Data were collected in four independent experiments with 2–4 donors per experiment. Similar analyses were performed for blood B cells from CIS patients (*n* = 18), RRMS patients (*n* = 19), and HC (D; *n* = 22), as well as low‐risk CIS (*n* = 13) and high‐risk CIS (*n* = 5) groups (E). B cells of CIS patients were sorted in nine independent experiments with 2–4 patients per experiment. B cells of HC and RRMS patients were sorted in 17 independent experiments, with 1–2 HC and 1–2 RRMS patients per experiment. MIF and 18S mRNA expression was measured in six individual experiments, with two independent experiments per patient group. Data are shown as mean ± SEM. Each dot represents the mean value of one individual, measured in duplicates. Student's *t*‐tests were used to compare groups. (F) Correlation between MIF mRNA levels and CXCR4/CD74 surface expression ratios in B cells from patients and healthy controls (*n* = 30). B cells were sorted, mRNA was measured in duplicates, and surface expression was analyzed in six independent experiments with 2 HC and 2–4 patients per experiment. *r* = Spearman's correlation; *p < 0.05, **p < 0.01, ****p < 0.0001.

MIF levels in both serum and plasma were not different between CIS patients and healthy controls (Fig. 4A and Supporting Information Fig. 3A) or high‐risk and low‐risk CIS subgroups (Fig. 4B and Supporting Information Fig. 3B), and did not correlate with CXCR4 and CD74 expression levels on B cells from the same individuals (Supporting Information Fig. 3C and D).

However, among blood immune subsets, MIF mRNA was predominantly expressed by B cells compared to paired T cells, monocytes and dendritic cells (Fig. 4C), but downregulated in B cells from RRMS patients versus HC (*p* < 0.01) and even further in B cells of CIS patients (CIS vs. HC, *p* < 0.0001; CIS vs. RRMS, *p* < 0.01; Fig. 4D). We found no differences in MIF mRNA levels between B cells from low‐risk and high‐risk CIS subgroups (Fig. 4E). Low MIF mRNA levels corresponded to high CXCR4/CD74 surface expression ratios in B cells of patients and controls (*p* = 0.005; Fig. 4F). This point to the existence of a disturbed regulation loop between MIF and MIF receptors in B cells of early MS patients.

### MIF, CD74, and CXCR4 are part of a reciprocal negative regulation loop in human B cells

**Figure 5:**
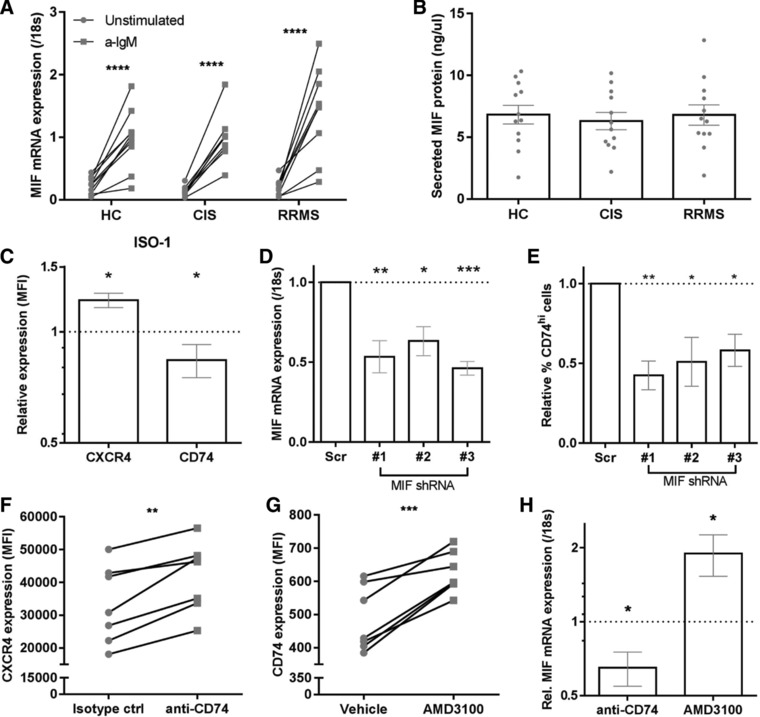
Interference with MIF signaling pathways reveals mutual regulation of CD74, CXCR4, and MIF expression in in vitro‐activated B cells. B cells sorted from CIS, RRMS, and HC blood (*n* = 9–12 per group) were activated in vitro for 24 h using anti‐IgM and analyzed for MIF expression (A; qPCR) and secretion into culture media (B; ELISA). Stimulations have been done in three individual experiments with 3–4 patients per group per experiment. Each dot represents the mean value of one individual, measured in duplicates. (C) In vitro‐activated B cells from healthy blood were treated with and without MIF inhibitor ISO‐1 for 24 h and assessed for CXCR4 and CD74 surface expression using FACS (*n* = 6). Stimulations have been done in three individual experiments with two controls per group per experiment. (D, E) The human B‐cell line Raji (CD74^hi^) was transfected with three distinct MIF shRNA constructs and compared with scrambled controls for MIF mRNA levels (D) and the percentage of CD74^hi^ cells (E) at day 3 (*n* = 5). Data were measured in five individual experiments with one set of scrambled and three different shRNA's per experiment. In vitro‐activated B cells were also evaluated for CXCR4 (F, *n* = 7), CD74 (G, *n* = 7) and MIF mRNA (H, *n* = 5–6, measured in duplicates) expression after 24 h treatment with anti‐CD74 (LN2) or isotype antibody (F, H) and CXCR4 antagonist AMD3100 (G, H). Stimulations have been done in three individual experiments with 2–3 controls per group per experiment. All used controls were set at 1 (dotted line). Data are shown as mean ± SEM. Paired *t*‐tests were performed to compare groups. *p < 0.05, **p < 0.01, ***p < 0.001.

In vitro, B cells showed enhanced MIF expression and secretion after activation with anti‐IgM, which was comparable between patients and controls (Fig. 5A and B). To determine potential crosstalk between MIF, CXCR4, and CD74, we used the specific MIF inhibitor ISO‐1 16 to block MIF‐mediated signaling in in vitro‐activated B cells. ISO‐1 treatment of these B cells resulted in a CXCR4 upregulation and CD74 downregulation (Fig. 5C), reflecting the inverse correlation between MIF and CXCR4/CD74 expression levels in B cells ex vivo (Fig. 4F). In parallel to this, in the human B‐cell line Raji, which abundantly expresses CD74 17, the percentage of CD74^hi^ cells decreased after MIF knockdown using three distinct shRNA constructs (Fig. 5D and E). This indicates that CXCR4 and CD74 surface expression is inversely regulated by endogenous MIF in B cells. After treatment with anti‐CD74 antibody (LN2), CXCR4 surface expression was increased, whereas MIF expression was reduced in in vitro‐activated B cells (Fig. 5F and H). Vice versa, activated B cells treated with CXCR4 antagonist AMD3100 18 showed increased CD74 and MIF levels (Fig. 5G and H). These data demonstrate that MIF, CD74, and CXCR4 expression in human B cells is tightly and mutually controlled.

### CXCR4/CD74 controls the inflammatory, proliferative, and Fas‐mediated apoptotic potential of B cells

**Figure 6:**
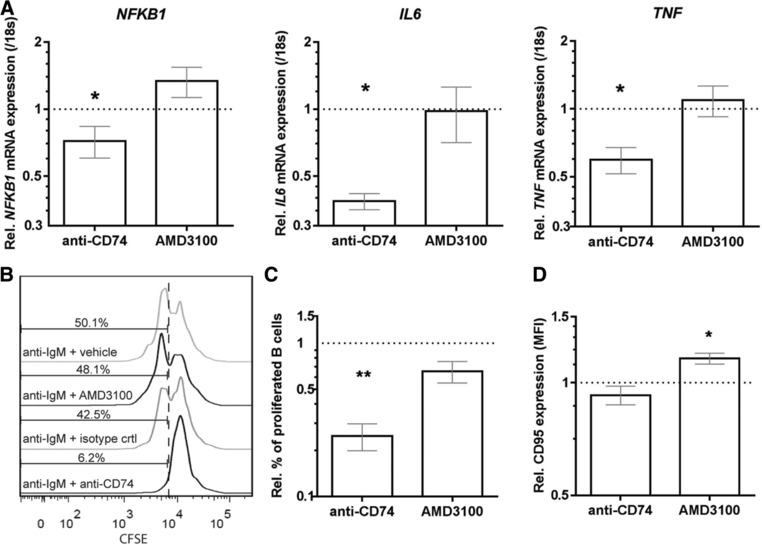
CD74 and CXCR4 on human B cells differentially control proinflammatory gene expression, proliferation, and sensitivity to Fas‐mediated apoptosis. B cells from healthy blood were in vitro‐activated with a‐IgM and subsequently treated with anti‐CD74 antibody (LN2) or AMD3100. Data were compared to their respective controls for relative NF‐κB, IL‐6, and TNF‐α mRNA expression after 24 h using qPCR (A, *n* = 4–6, measured in duplicates), as well as CFSE‐based proliferation (B and C, *n* = 5) and Fas (CD95) surface levels (D, *n* = 5) after 3 days using FACS. Stimulations have been done in three individual experiments with two controls per group per experiment. All used controls were set at 1 (dotted line). Data are shown as mean ± SEM. Paired *t*‐tests were performed to compare groups. *p < 0.05, **p < 0.01.

To determine how the reciprocal negative regulation of CD74 and CXCR4 is associated with the function of B cells, we compared the proinflammatory, proliferative, and survival capacity of in vitro‐activated B cells before and after blocking of these MIF receptors. Treatment of these B cells with anti‐CD74 antibody LN2 (24 h) suppressed the induction of proinflammatory genes *NFKB1, IL6*, and *TNF* (Fig. 6A). This was not found in in vitro‐activated B cells treated with CXCR4 antagonist AMD3100 (Fig. 6A). These differences were verified on protein level (Supporting Information Fig. 4). Also B‐cell proliferation was inhibited after treatment for 3 days with LN2 antibody and not with AMD3100, as determined by CFSE labeling (Fig. 6B and C). Finally, surface expression of the death receptor Fas (CD95) was triggered in AMD3100‐ and not in LN2‐treated B cells after 3 days of activation (Fig. 6D). These results imply that in humans, CD74 primarily boosts the proinflammatory and proliferative capacity of B cells, while CXCR4 makes B cells less sensitive for Fas‐mediated apoptosis 19.

## Discussion

The aim of this study was to elucidate the impact of the B‐cell intrinsic MIF pathway on early disease onset in MS patients. We demonstrate that decreased CD74 and increased CXCR4 expression on B cells in blood are associated with early MS diagnosis. This was shown for CIS patients who will rapidly develop MS as well as for clinically definite MS patients. In vitro experiments supported the inverse regulation of MIF/CD74 and CXCR4 expression in B cells ex vivo, which differentially controlled their proinflammatory capacity, proliferation, and sensitivity to Fas‐mediated apoptosis (Supporting Information Fig. 5). The observed CXCR4^hi^CD74^lo^ B‐cell phenotype in early MS blood points to the presence of more immature B‐cell populations with senescent features that survived peripheral tolerance checkpoints in MS 3.

There are several lines of evidence implying that CD74 downregulation and CXCR4 upregulation disrupt the selection of immature B cells. During B‐cell development, “new emigrant” transitional subsets in blood are negatively selected in secondary lymphoid organs for autoreactive clones before developing into naive mature subsets. B cells in CD74‐deficient mice revealed an arrest at the transitional stage 20 and a reduced lifespan 21. Consistently, decreased CD74 expression impaired B‐cell maturation in patients with X‐linked lymphoproliferative disease 22. Both cell autonomous and nonautonomous roles of CD74 could explain these defects in B‐cell development. As a cell surface receptor, CD74 triggers B‐cell proliferation in mice 8 and humans (current study), supporting the lowered proliferative capacity of naive (CD74^lo^) versus memory (CD74^hi^) populations 23. The reduced expression of CD74 on B cells in early MS blood thus might reflect a functional state of anergy, contributing to the persistence of pathogenic immature B cells in the periphery 24. This is underlined by the downmodulation of proinflammatory cytokines TNF‐α and IL‐6, as well as MIF after blockade of CD74 on B cells. Alternatively, defective processing of CD74 results in the accumulation of an N‐terminal fragment, which interferes with B‐cell receptor signaling to suppress B‐cell maturation in an MHC class II‐independent manner 9, 25, 26. In addition to these cell‐intrinsic effects, a loss of CD74 can also influence T helper cell‐mediated selection of naive mature B cells via altered MHC class II antigen presentation 27.

In contrast to CD74, CXCR4 was found to be the most abundant on naive B cells, which controls their development in germinal centers. CXCR4^hi^ B cells are localized in the dark zone to undergo somatic hypermutation, whereas antigen‐ and T helper cell‐based selection of CXCR4^lo^ B cells occurs in the light zone 28, 29. Our data show that blocking of CXCR4 signaling in B cells increases CD95 (Fas) expression, which is essential for the elimination of autoreactive clones by T helper cells 30. Hence, it may be speculated that overexpression of CXCR4 on B cells as observed in early MS results in the escape of naive populations from T helper cell‐based selection in the light zone, via reduced Fas expression and enhanced migration to the dark zone. Central tolerance checkpoints were not defective in MS patients 3, making it unlikely that the abundance of CXCR4 affects precursor B‐cell selection in the bone marrow 31. In vivo studies need to be performed in the future to confirm these roles of CD74 and CXCR4 in peripheral B‐cell tolerance in MS.

To our knowledge, the coregulation of MIF, CD74, and CXCR4 in human B cells and MS patients has never been studied before. Although abundantly expressed by B cells compared to other immune populations, MIF is downregulated in blood B cells from early MS patients. This downregulation links to the survival of autoreactive naive B cells, as seen in atherosclerotic mice 13 and MS 3, and coincides with decreased CD74 and increased CXCR4 surface expression, as part of a tightly controlled regulation loop in B cells. The reciprocal expression of CD74 and CXCR4 was supported by the increased migration capacity of B cells toward CXCL12 after inhibition of CD74 12, 32. Since MIF has a higher affinity for CD74 than for CXCR4 7, 10, a possible underlying mechanism of this reciprocal expression is that MIF‐mediated endocytosis of CD74 results in interaction with the adaptor molecule β‐arrestin, thereby preventing binding to and internalization of CXCR4 33, 34, 35 (Supporting Information Fig. 5).

Extracellular MIF levels were not different in the blood of early MS patients. However, previous studies showed increased levels of MIF in MS CSF 36 as well as in MS lesions and not normal‐appearing white matter 37. This suggests that local MIF production predominantly attracts CXCR4^hi^ B cells from the blood 10, 12 to mediate early MS disease activity. Another CXCR4 ligand, CXCL12, was also found to be abundant in MS CSF, but did not correlate to local B‐cell infiltration and activation 38. After recruitment to the CNS, CXCR4^hi^CD74^lo^ B cells will probably be activated, resulting in increased CD74 expression and MIF production. CD74 triggering by autocrine MIF may then enhance their ability to proliferate, as shown for tumor cells 39, and produce proinflammatory cytokines to mediate CNS pathology in MS.

This study shows that MIF and MIF receptors CD74 and CXCR4 are coordinately expressed in B cells to control their inflammatory, proliferative, and apoptotic potential in humans. The dysregulation of this B cell‐intrinsic loop in early MS pleads for future studies on the processing and cooperation of CD74 and CXCR4 during autoreactive B‐cell development. Also more insights into the effects of autocrine and T helper cell‐derived MIF on their development will lead to better understanding of the role of B cells as central players in MS and other autoimmune diseases.

## Materials and Methods

### Patients

Patient characteristics are summarized in Supporting Information Table 1. All clinically isolated syndrome (CIS) and relapsing‐remitting MS (RRMS) patients as well as healthy controls (HC) were included in MS Center ErasMS at Erasmus MC (Rotterdam, The Netherlands). CIS was defined as a first clinical attack of demyelination in the CNS 14. Clinically definite MS (CDMS) was diagnosed when a patient experienced two attacks with clinical evidence of two separate lesions according to the Poser criteria 40. CIS patients were sampled within 4 months after their first attack. From our prospective cohort, we selected CIS patients who did not develop CDMS for at least 5 years of follow‐up (low‐risk CIS) and CIS patients who were diagnosed with CDMS within 1 year after CIS diagnosis (high‐risk CIS). Fatigue severity was assessed using Krupp's fatigue severity scale 41. RRMS patients were diagnosed according to the McDonalds criteria 42 and were age‐ and gender‐matched with healthy control subjects. For functional studies, buffy coats (Sanquin, Amsterdam, The Netherlands) were obtained from healthy volunteers. All patients and controls gave written informed consent and study protocols were approved by the medical ethics committee of the Erasmus MC.

### Peripheral blood sampling

PBMCs and plasma were isolated from whole blood with the use of CPT^TM^ heparin tubes, while serum was isolated using coagulation tubes (both BD Biosciences, San Jose, CA). Samples were processed according to the manufacturer's instructions. PBMCs were stored in liquid nitrogen; plasma and serum were stored in –80°C until analysis.

### Human MIF ELISA

ELISA was used to measure human MIF levels in serum and plasma using the four‐span approach, as previously described 43. In brief, anti‐human MIF polyclonal antibodies raised in chicken and rabbit were used as capture and trapping antibodies. Microtiter plates were coated with a duck anti‐chicken antibody. A HRP‐labeled goat anti‐rabbit antibody was used for the detection. Distinct concentrations of rhMIF (R&D Systems, Minneapolis, MN) were used to generate a standard curve. The analytic sensitivity of the human MIF ELISA was 39 pg/mL and the coefficients of variation were 6% for intrarun and 12% for interrun.

### Flow cytometry and cell sorting

In‐depth flow cytometric analysis of B cells were performed using anti‐human monoclonal antibodies against CD3, CD19, CD24, CD27, CD38, CD69, CD74, CD95, CXCR4, HLA‐DR, IgA, IgD, IgG, and IgM. Details of these antibodies are indicated in Supporting Information Table 2. All measurements were performed on an LSRFortessa^TM^ flow cytometer and data were analyzed using FACSDiva 8.1 software (both BD Biosciences). Guidelines for the use of flow cytometry in immunological studies have been followed 44.

### Intracellular cytokine staining

Cells were stimulated for 5 h using phorbol 12‐myristate 13‐acetate (PMA; 20 ng/mL) and ionomycin (500 ng/mL, both Sigma‐Aldrich), in the presence of BD GolgiStop^TM^. Stimulated cells were stained with BD Horizon™ Fixable Viability Stain 700, fixed and permeabilized using BD Cytofix/Cytoperm^TM^ according to the provided protocol and stained for IL‐6 and TNF‐α (Supporting Information Table 2, BD Biosciences).

### RNA isolation and quantitative PCR

CD19^+^ B cells, CD3^+^ T cells, CD14^+^ monocytes, and CD56^−^HLA‐DR^+^ dendritic cells were sorted using a high‐speed cell sorter (FACSAria III^TM^; BD Biosciences), resulting in a purity of more than 95%. Subsequently, mRNA was isolated using GenElute^TM^ Mammalian RNA Kit (Sigma‐Aldrich, St. Louis, MO) and reversely transcribed into cDNA following a standard laboratory protocol with the use of SuperScript II^®^ Reverse Transcriptase (Invitrogen, Paisley, UK). Primers and probes were selected by using the Universal Probe Library Assay Design Centre (Roche Applied Science, Penzberg, Germany). To determine target gene mRNA expression levels, qPCR was performed using an Applied Biosystems 7900 Sequence Detector, which was programmed for the initial step of 2 min at 50°C and 10 min 95°C, followed by 40 thermal cycles of 15s at 95°C and 1 min at 60°C. For the calculation of relative mRNA levels, CT values per gene were related to standard curves, which were generated for each gene of interest. The 18S levels were measured as a control to normalize for RNA input. Primer sequences are listed in Supporting Information Table 3.

### In vitro activation and modulation of B cells

CD19^+^ B cells of healthy donors were isolated untouched by depleting all other cell types from PBMCs using the B‐cell isolation kit II and MACS (Miltenyi Biotec, Bergisch Gladbach, Germany). CD19^+^ B cells of patients and healthy controls were sorted using FACS and were cultured in RPMI supplemented with 10% fetal calf serum and 1% penicillin/streptavidin for 24 hours or 3 days with various stimuli; anti‐IgM (F(ab’)_2,_ 10 μg/mL, Jackson ImmunoResearch Inc., West Grove, PA) with or without MIF inhibitor ISO‐1 (100 μM, R&D Systems), a neutralizing anti‐human CD74 antibody (LN2; 10 μg/mL, BD Biosciences) or AMD3100 (10 μg/mL, Sigma‐Aldrich). Proliferation rates were addressed by labeling B cells with CFSE (eBioscience, San Diego, CA) before in vitro activation. For MIF knockdown, the human B‐cell line Raji was transfected with MIF shRNA‐containing pLKO.1 constructs (MISSION® shRNA Library, Sigma‐Aldrich) using Nucleofector Kit V from Lonza (Basel, Switzerland). Three different MIF shRNA constructs were used: #1 GACAGGGTCTACATCAACTAT, #2 CTACATCAACTATTACGACAT, and #3 CCTGCACAGCATCGGCAAGAT. MIF mRNA and CD74 surface expression was analyzed and compared to scrambled shRNA controls at day 3.

### Western blotting

Cells were lysed in radio‐immunoprecipitation assay lysis buffer supplemented with 10% complete protease inhibitor cocktail (Roche, Mannheim, Germany) on ice for 30 min, centrifuged at 4°C for 10 min at 10 000 x *g*. Cell lysates were reduced with 10% 2‐mercaptoethanol, denatured for 5min at 95^○^C, loaded onto a 10% precast polyacrylamide gel (Bio‐Rad, Hercules, CA) followed by immunoblotting on a Immobilon‐P membrane (Merck Millipore, Darmstadt, Germany) for 1 hours at 4°C. Membranes were blocked in 5% nonfat dry milk and incubated with rabbit anti‐human NF‐κB1 p105/p50 (D7H5M, Cell signaling technology, Danvers, MA) or mouse anti‐human β‐actin (AC‐15; Abcam, Cambridge, UK) and HRP‐conjugated swine‐anti‐rabbit Ig or goat‐anti‐mouse Ig (Dako, Glostrup, Denmark). Protein bands were visualized using Western Lightning Plus‐ECL (Perkin Elmer Inc., Waltham, MA).

### Statistical analyses

Statistical analyses were performed using Graphpad Prism 7 (GraphPad Software Inc., San Diego, CA). Data are expressed as mean ± SEM. Datasets were tested for normal distribution. Two‐tailed *t* tests or ANOVA were used to compare groups. Correlations between two parameters were tested by using Pearson's or Spearman correlation coefficients. *p*‐values < 0.05 were considered as statistically different.

## Author contributions

L.R., M.M., M.S., J.L., A.F.W., and J.M.H. conducted the experiments. L.R., R.Q.H., and M.M.L. designed the experiments. L.R. and M.M.L. wrote the paper. R.M.V. collected clinical samples and patient information. F.C.S. and A.J.G. provided and performed the MIF ELISA. M.M.L., and R.Q.H. acquired funding and supervised the project. All authors have read the paper and gave feedback.

## Conflict of interests

The authors declare no commercial or financial conflict of interests.

**Abbreviations**
CIS: clinically isolated syndrome
CDMS: clinically definite MS
HC: healthy control
RRMS: relapsing‐remitting MS

## Supporting information

Supporting information

Supplementary Figure 1: Reproducibility and validation of the CXCR4hiCD74lo B‐cell phenotype in early MS patients.
Supplementary Figure 2: CXCR4/CD74 expression ratios on distinct B‐cell subsets in RRMS versus HC blood.
Supplementary Figure 3: Circulating MIF levels and their association with CXCR4/CD74 expression ratios on blood B cells in CIS.
Supplementary Figure 4: Effects of CD74 and CXCR4 blocking on NF‐κB1, IL‐6 and TNF‐α protein expression in primary B cells
Supplementary Figure 5: Potential counter‐regulatory mechanism and downstream effects of CD74 and CXCR4 signaling in peripheral B cells of early MS patients.
Supplementary Table 1: Clinical information of patients and healthy controls
Supplementary Table 2: Monoclonal antibodies used for FACS
Supplementary Table 3: Primer sequences used for real‐time PCR

## References

[1] Nylander, A. and Hafler, D. A. , Multiple sclerosis. J. Clin. Invest. 2012 122: 1180–1188. 22466660 10.1172/JCI58649 PMC3314452

[2] Hauser, S. L. , Waubant, E. , Arnold, D. L. , Vollmer, T. , Antel, J. , Fox, R. J. , Bar‐Or, A. et al., B‐cell depletion with rituximab in relapsing‐remitting multiple sclerosis. N. Engl. J. Med. 2008 358: 676–688. 18272891 10.1056/NEJMoa0706383

[3] Kinnunen, T. , Chamberlain, N. , Morbach, H. , Cantaert, T. , Lynch, M. , Preston‐Hurlburt, P. , Herold, K. C. et al., Specific peripheral B cell tolerance defects in patients with multiple sclerosis. J. Clin. Invest. 2013 123: 2737–2741. 23676463 10.1172/JCI68775 PMC3668812

[4] Calandra, T. and Roger, T. , Macrophage migration inhibitory factor: a regulator of innate immunity. Nat. Rev. Immunol. 2003 3: 791–800. 14502271 10.1038/nri1200 PMC7097468

[5] Baker, D. , O'Neill, J. K. and Turk, J. L. , Cytokines in the central nervous system of mice during chronic relapsing experimental allergic encephalomyelitis. Cell. Immunol. 1991 134: 505–510. 1902401 10.1016/0008-8749(91)90321-2

[6] Denkinger, C. M. , Denkinger, M. , Kort, J. J. , Metz, C. and Forsthuber, T. G. , In vivo blockade of macrophage migration inhibitory factor ameliorates acute experimental autoimmune encephalomyelitis by impairing the homing of encephalitogenic T cells to the central nervous system. J. Immunol. 2003 170: 1274–1282. 12538686 10.4049/jimmunol.170.3.1274

[7] Leng, L. , Metz, C. N. , Fang, Y. , Xu, J. , Donnelly, S. , Baugh, J. , Delohery, T. et al., MIF signal transduction initiated by binding to CD74. J. Exp. Med. 2003 197: 1467–1476. 12782713 10.1084/jem.20030286 PMC2193907

[8] Starlets, D. , Gore, Y. , Binsky, I. , Haran, M. , Harpaz, N. , Shvidel, L. , Becker‐Herman, S. et al., Cell‐surface CD74 initiates a signaling cascade leading to cell proliferation and survival. Blood 2006 107: 4807–4816. 16484589 10.1182/blood-2005-11-4334

[9] Matza, D. , Lantner, F. , Bogoch, Y. , Flaishon, L. , Hershkoviz, R. and Shachar, I. , Invariant chain induces B cell maturation in a process that is independent of its chaperonic activity. Proc. Natl. Acad. Sci. USA 2002 99: 3018–3023. 11867743 10.1073/pnas.052703299 PMC122465

[10] Bernhagen, J. , Krohn, R. , Lue, H. , Gregory, J. L. , Zernecke, A. , Koenen, R. R. , Dewor, M. et al., MIF is a noncognate ligand of CXC chemokine receptors in inflammatory and atherogenic cell recruitment. Nat. Med. 2007 13: 587–596. 17435771 10.1038/nm1567

[11] Schwartz, V. , Lue, H. , Kraemer, S. , Korbiel, J. , Krohn, R. , Ohl, K. , Bucala, R. et al., A functional heteromeric MIF receptor formed by CD74 and CXCR4. FEBS Lett. 2009 583: 2749–2757. 19665027 10.1016/j.febslet.2009.07.058 PMC2911026

[12] Klasen, C. , Ohl, K. , Sternkopf, M. , Shachar, I. , Schmitz, C. , Heussen, N. , Hobeika, E. et al., MIF promotes B cell chemotaxis through the receptors CXCR4 and CD74 and ZAP‐70 signaling. J. Immunol. 2014 192: 5273–5284. 24760155 10.4049/jimmunol.1302209

[13] Schmitz, C. , Noels, H. , El Bounkari, O. , Straussfeld, E. , Megens, R. T. A. , Sternkopf, M. , Alampour‐Rajabi, S. et al., Mif‐deficiency favors an atheroprotective autoantibody phenotype in atherosclerosis. FASEB J. 2018. 32:4428–4443. 29543531 10.1096/fj.201800058R PMC6207164

[14] Miller, D. H. , Chard, D. T. and Ciccarelli, O. , Clinically isolated syndromes. Lancet Neurol. 2012 11: 157–169. 22265211 10.1016/S1474-4422(11)70274-5

[15] Runia, T. F. , Jafari, N. , Siepman, D. A. and Hintzen, R. Q. , Fatigue at time of CIS is an independent predictor of a subsequent diagnosis of multiple sclerosis. J. Neurol. Neurosurg. Psychiatry. 2015 86: 543–546. 25053770 10.1136/jnnp-2014-308374

[16] Al‐Abed, Y. , Dabideen, D. , Aljabari, B. , Valster, A. , Messmer, D. , Ochani, M. , Tanovic, M. et al., ISO‐1 binding to the tautomerase active site of MIF inhibits its pro‐inflammatory activity and increases survival in severe sepsis. J. Biol. Chem. 2005 280: 36541–36544. 16115897 10.1074/jbc.C500243200

[17] Wraight, C. J. , van Endert, P. , Moller, P. , Lipp, J. , Ling, N. R. , MacLennan, I. C. , Koch, N. et al., Human major histocompatibility complex class II invariant chain is expressed on the cell surface. J. Biol. Chem. 1990 265: 5787–5792. 1690714

[18] Hatse, S. , Princen, K. , Bridger, G. , De Clercq, E. and Schols, D. , Chemokine receptor inhibition by AMD3100 is strictly confined to CXCR4. FEBS Lett. 2002 527: 255–262. 12220670 10.1016/s0014-5793(02)03143-5

[19] Koncz, G. and Hueber, A. O. , The Fas/CD95 receptor regulates the death of autoreactive B cells and the selection of antigen‐specific B cells. Front Immunol. 2012 3: 207. 22848207 10.3389/fimmu.2012.00207 PMC3404404

[20] Shachar, I. and Flavell, R. A. , Requirement for invariant chain in B cell maturation and function. Science 1996 274: 106–108. 8810244 10.1126/science.274.5284.106

[21] Benlagha, K. , Park, S. H. , Guinamard, R. , Forestier, C. , Karlsson, L. , Chang, C. H. and Bendelac, A. , Mechanisms governing B cell developmental defects in invariant chain‐deficient mice. J. Immunol. 2004 172: 2076–2083. 14764672 10.4049/jimmunol.172.4.2076

[22] Radomir, L. , Cohen, S. , Kramer, M. P. , Bakos, E. , Lewinsky, H. , Barak, A. , Porat, Z. et al., T cells regulate peripheral naive mature B cell survival by cell‐cell contact mediated through SLAMF6 and SAP. J. Immunol. 2017 199: 2745–2757. 28904129 10.4049/jimmunol.1700557 PMC5805483

[23] Allman, D. , Lindsley, R. C. , DeMuth, W. , Rudd, K. , Shinton, S. A. and Hardy, R. R. , Resolution of three nonproliferative immature splenic B cell subsets reveals multiple selection points during peripheral B cell maturation. J. Immunol. 2001 167: 6834–6840. 11739500 10.4049/jimmunol.167.12.6834

[24] Yarkoni, Y. , Getahun, A. and Cambier, J. C. , Molecular underpinning of B‐cell anergy. Immunol. Rev. 2010 237: 249–263. 20727040 10.1111/j.1600-065X.2010.00936.x PMC2968701

[25] Schneppenheim, J. , Loock, A. C. , Huttl, S. , Schweizer, M. , Lullmann‐Rauch, R. , Oberg, H. H. , Arnold, P. et al., The influence of MHC class II on B cell defects induced by invariant chain/CD74 N‐terminal fragments. J. Immunol. 2017 199: 172–185. 28550201 10.4049/jimmunol.1601533

[26] Huttl, S. , Klasener, K. , Schweizer, M. , Schneppenheim, J. , Oberg, H. H. , Kabelitz, D. , Reth, M. et al., Processing of CD74 by the intramembrane protease SPPL2a is critical for B cell receptor signaling in transitional B cells. J. Immunol. 2015 195: 1548–1563. 26157172 10.4049/jimmunol.1403171

[27] Yeh, C.‐H. , Nojima, T. , Kuraoka, M. and Kelsoe, G. H. , The limit of MHC class II‐driven selection in germinal centers. J. Immunol. 2017 198: 152.114–152.114.

[28] Allen, C. D. , Ansel, K. M. , Low, C. , Lesley, R. , Tamamura, H. , Fujii, N. and Cyster, J. G. , Germinal center dark and light zone organization is mediated by CXCR4 and CXCR5. Nat. Immunol. 2004 5: 943–952. 15300245 10.1038/ni1100

[29] Victora, G. D. , Dominguez‐Sola, D. , Holmes, A. B. , Deroubaix, S. , Dalla‐Favera, R. and Nussenzweig, M. C. , Identification of human germinal center light and dark zone cells and their relationship to human B‐cell lymphomas. Blood 2012 120: 2240–2248. 22740445 10.1182/blood-2012-03-415380 PMC3447782

[30] Rathmell, J. C. , Cooke, M. P. , Ho, W. Y. , Grein, J. , Townsend, S. E. , Davis, M. M. and Goodnow, C. C. , CD95 (Fas)‐dependent elimination of self‐reactive B cells upon interaction with CD4+ T cells. Nature 1995 376: 181–184. 7603571 10.1038/376181a0

[31] Nie, Y. , Waite, J. , Brewer, F. , Sunshine, M. J. , Littman, D. R. and Zou, Y. R. , The role of CXCR4 in maintaining peripheral B cell compartments and humoral immunity. J. Exp. Med. 2004 200: 1145–1156. 15520246 10.1084/jem.20041185 PMC2211858

[32] Frolich, D. , Blassfeld, D. , Reiter, K. , Giesecke, C. , Daridon, C. , Mei, H. E. , Burmester, G. R. et al., The anti‐CD74 humanized monoclonal antibody, milatuzumab, which targets the invariant chain of MHC II complexes, alters B‐cell proliferation, migration, and adhesion molecule expression. Arthritis Res. Ther. 2012 14: R54. 22404985 10.1186/ar3767 PMC3446420

[33] Orsini, M. J. , Parent, J. L. , Mundell, S. J. , Marchese, A. and Benovic, J. L. , Trafficking of the HIV coreceptor CXCR4. Role of arrestins and identification of residues in the c‐terminal tail that mediate receptor internalization. J. Biol. Chem. 1999 274: 31076–31086. 10521508 10.1074/jbc.274.43.31076

[34] Xie, L. , Qiao, X. , Wu, Y. and Tang, J. , beta‐Arrestin1 mediates the endocytosis and functions of macrophage migration inhibitory factor. PLoS One 2011 6: e16428. 21283538 10.1371/journal.pone.0016428 PMC3026819

[35] van der Vorst, E. P. , Doring, Y. and Weber, C. , Chemokines and their receptors in Atherosclerosis. J. Mol. Med. 2015 93: 963–971. 26175090 10.1007/s00109-015-1317-8 PMC4577534

[36] Niino, M. , Ogata, A. , Kikuchi, S. , Tashiro, K. and Nishihira, J. , Macrophage migration inhibitory factor in the cerebrospinal fluid of patients with conventional and optic‐spinal forms of multiple sclerosis and neuro‐Behcet's disease. J. Neurol. Sci. 2000 179: 127–131. 11054496 10.1016/s0022-510x(00)00397-x

[37] Cox, G. M. , Kithcart, A. P. , Pitt, D. , Guan, Z. , Alexander, J. , Williams, J. L. , Shawler, T. et al., Macrophage migration inhibitory factor potentiates autoimmune‐mediated neuroinflammation. J. Immunol. 2013 191: 1043–1054. 23797673 10.4049/jimmunol.1200485

[38] Krumbholz, M. , Theil, D. , Cepok, S. , Hemmer, B. , Kivisakk, P. , Ransohoff, R. M. , Hofbauer, M. et al., Chemokines in multiple sclerosis: CXCL12 and CXCL13 up‐regulation is differentially linked to CNS immune cell recruitment. Brain 2006 129: 200–211. 16280350 10.1093/brain/awh680

[39] Verjans, E. , Noetzel, E. , Bektas, N. , Schutz, A. K. , Lue, H. , Lennartz, B. , Hartmann, A. et al., Dual role of macrophage migration inhibitory factor (MIF) in human breast cancer. BMC Cancer 2009 9: 230. 19602265 10.1186/1471-2407-9-230 PMC2716369

[40] Poser, C. M. , Paty, D. W. , Scheinberg, L. , McDonald, W. I. , Davis, F. A. , Ebers, G. C. , Johnson, K. P. et al., New diagnostic criteria for multiple sclerosis: guidelines for research protocols. Ann. Neurol. 1983 13: 227–231. 6847134 10.1002/ana.410130302

[41] Krupp, L. B. , LaRocca, N. G. , Muir‐Nash, J. and Steinberg, A. D. , The fatigue severity scale. Application to patients with multiple sclerosis and systemic lupus erythematosus. Arch. Neurol. 1989 46: 1121–1123. 2803071 10.1001/archneur.1989.00520460115022

[42] Polman, C. H. , Reingold, S. C. , Banwell, B. , Clanet, M. , Cohen, J. A. , Filippi, M. , Fujihara, K. et al., Diagnostic criteria for multiple sclerosis: 2010 revisions to the McDonald criteria. Ann. Neurol. 2011 69: 292–302. 21387374 10.1002/ana.22366 PMC3084507

[43] Radstake, T. R. , Sweep, F. C. , Welsing, P. , Franke, B. , Vermeulen, S. H. , Geurts‐Moespot, A. , Calandra, T. et al., Correlation of rheumatoid arthritis severity with the genetic functional variants and circulating levels of macrophage migration inhibitory factor. Arthritis Rheum. 2005 52: 3020–3029. 16200611 10.1002/art.21285

[44] Cossarizza, A. , Chang, H. D. , Radbruch, A. , Akdis, M. , Andra, I. , Annunziato, F. , Bacher, P. et al., Guidelines for the use of flow cytometry and cell sorting in immunological studies. Eur. J. Immunol. 2017 47: 1584–1797. 29023707 10.1002/eji.201646632 PMC9165548

