# Supplementary material for: The macrophage migration inhibitory factor pathway in human B cells is tightly controlled and dysregulated in multiple sclerosis
Source: Eur J Immunol. 2018 Sep 25;48(11):1861–71. doi: 10.1002/eji.201847623 (PMC6282801; doi:10.1002/eji.201847623)
Supplement: Supplementary file 2 — Supplementary Figure 1: Reproducibility and validation of the CXCR4hiCD74lo B‐cell phenotype in early MS patients. Supplementary Figure 2: CXCR4/CD74 expression ratios on distinct B‐cell subsets in RRMS versus HC blood. Supplementary Figure 3: Circulating MIF levels and their association with CXCR4/CD74 expression ratios on blood B cells in CIS. Supplementary Figure 4: Effects of CD74 and CXCR4 blocking on NF‐κB1, IL‐6 and TNF‐α protein expression in primary B cells Supplementary Figure 5: Potential counter‐regulatory mechanism and downstream effects of CD74 and CXCR4 signaling in peripheral B cells of early MS patients. Supplementary Table 1: Clinical information of patients and healthy controls Supplementary Table 2: Monoclonal antibodies used for FACS Supplementary Table 3: Primer sequences used for real‐time PCR [file EJI-48-1861-s002.pdf]

European Journal of Immunology

Supporting Information
for

DOI 10.1002/eji.201847623

Liza Rijvers, Marie-José Melief, Roos M. van der Vuurst de Vries, Maeva Stéphant, Jamie van Langelaar, Annet F. Wierenga-Wolf, Jeanet M. Hogervorst, Anneke J. Geurts-Moespot, Fred C. G. J. Sweep, Rogier Q. Hintzen and Marvin M. van Luijn

The macrophage migration inhibitory factor pathway in human B cells is tightly controlled and dysregulated in multiple sclerosis

© 2018 The Authors. *European Journal of Immunology* published by WILEY-VCH Verlag GmbH & Co. KGaA, Weinheim.

Supplementary Data

Supplementary Figure 1

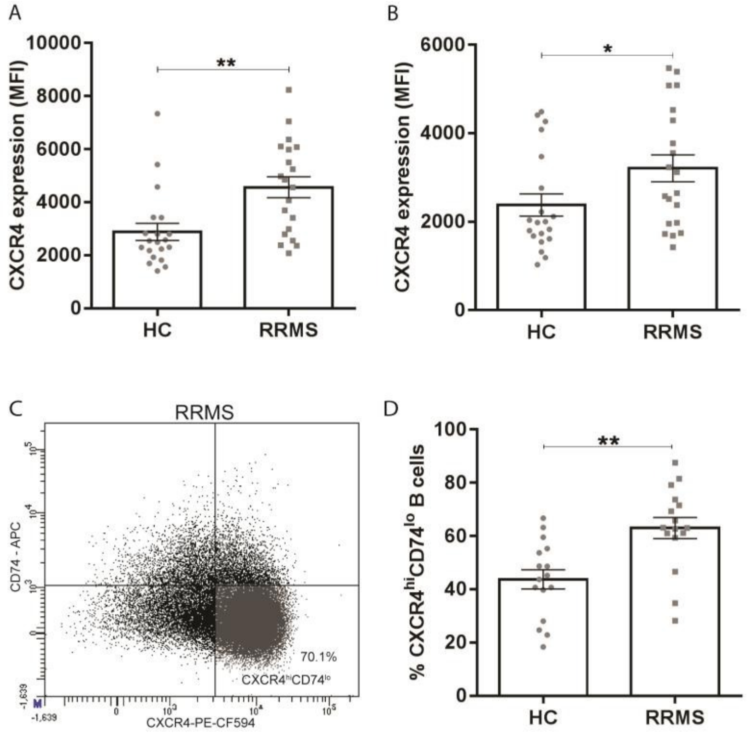

Supplementary Figure 1: Reproducibility and validation of the CXCR4^{hi}CD74^{lo} B-cell phenotype in early MS patients.

(A) Blood B cells of RRMS patients (n=20) and HC (n=20) from the screening cohort (see Supplementary Table 1) were reassessed for CXCR4 expression levels using the 12G5 antibody labeled with APC instead of PE-CF594. The latter antibody was used for the FACS analysis in Fig. 1B and 1D. Similar experiments were performed for B cells from an additional cohort of RRMS patients (n=20) and matched HC (n=20, see Supplementary Table 1; **B**). Data were obtained from 10 individual experiments, with B cells from 2 HC and 2 RRMS patients analyzed per experiment. **(C-D)** Representative dotplot and quantification of CXCR4^{hi}CD74^{lo} frequencies of B cells from RRMS patients and HC (screening cohort, see Supplementary Table 1). Data are shown as mean ± SEM. Student's t-tests were used to compare the groups. * $p<0.05$, ** $p<0.01$.

Supplementary Figure 2

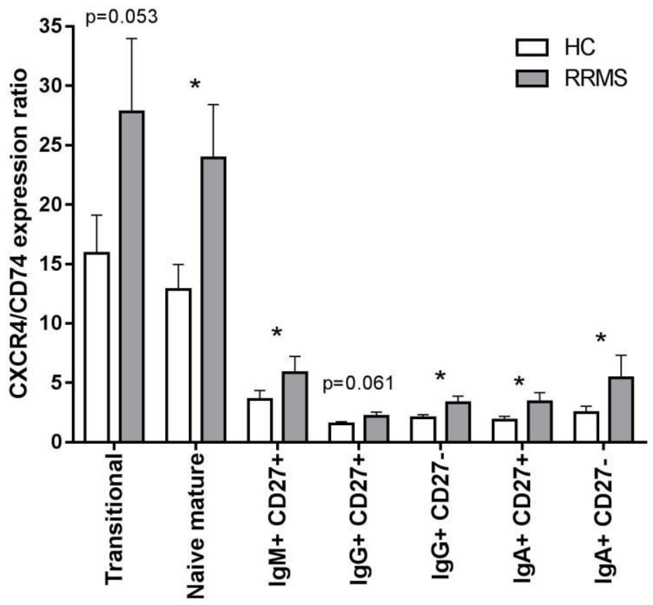

Supplementary Figure 2: CXCR4/CD74 expression ratios on distinct B-cell subsets in RRMS versus HC blood.

Gating of the subsets is shown in Fig. 3. Data were obtained from 3 individual FACS experiments, with B cells from 5 HC and 5 RRMS patients analyzed per experiment. Data are shown as mean ± SEM. Mann-Whitney U tests were used to compare subsets between the RRMS and HC groups. * $p<0.05$.

Supplementary Figure 3

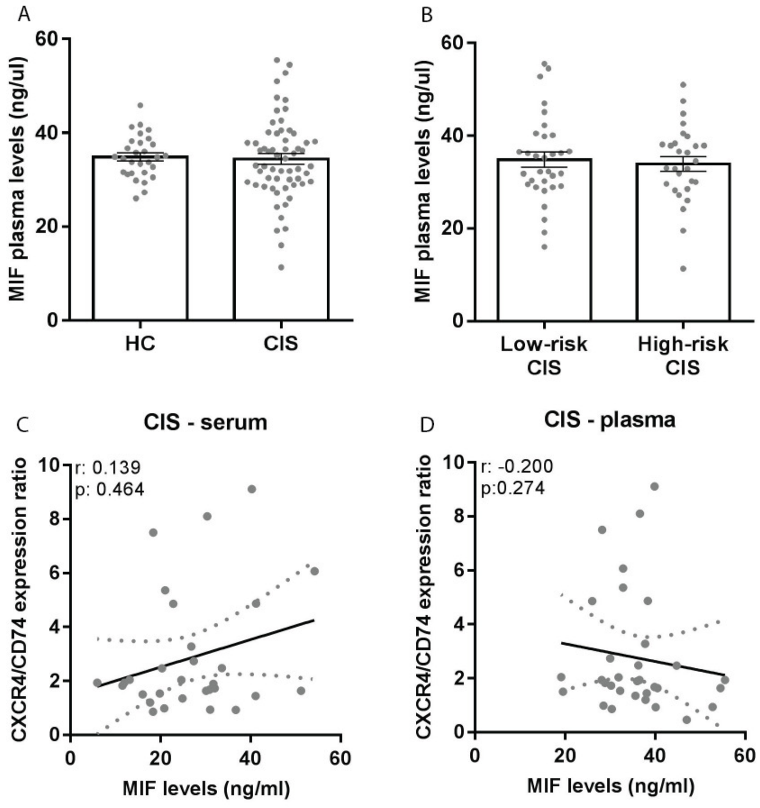

Supplementary Figure 3: Circulating MIF levels and their association with CXCR4/CD74 expression ratios on blood B cells in CIS.

MIF plasma levels (ng/µl) were compared between CIS patients (n=60) and HC (n=29; **A**), as well as low-risk CIS (n=32) and high-risk CIS (n=28; **B**) subgroups using ELISA. Data is measured in one single experiment. Each dot represents the mean value of one individual, measured in duplicates. Data are shown as mean ± SEM. The presence of MIF in serum **(C)** and plasma **(D)** was correlated to CXCR4/CD74 expression ratios on B cells in CIS blood (Fig. 2C). No significant differences and correlations were found.

Supplementary Figure 4

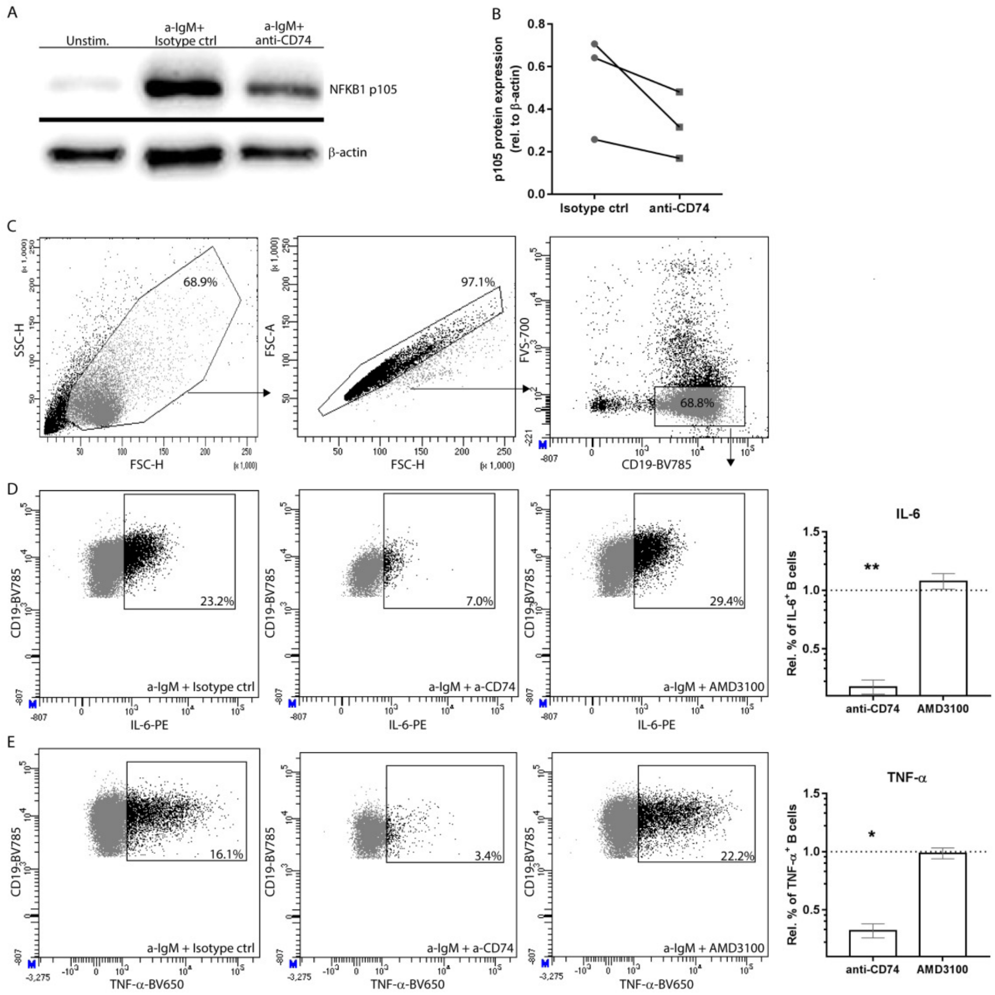

Supplementary Figure 4: Effects of CD74 and CXCR4 blocking on NF-κB1, IL-6 and TNF-α protein expression in primary B cells

(A-B) Representative image **(A)** and quantification **(B)** of NF-κB1 protein p105 expression in untreated as well anti-CD74 (LN2) or isotype control treated B cells from healthy blood after 24h of a-IgM stimulation (n=3, measured in one experiment). NF-κB1 p105 protein levels were normalized based on β-actin expression using the same blot. **(C-E)** IL-6 And TNF-α protein expression in anti-CD74 antibody (LN2) or AMD3100 treated healthy blood after in vitro-activation with a-IgM for 72h. Data was compared to their respective controls (n=3, measured in one experiment). **(C)** Gating of viable CD19+ B cells. **(D-E)** Gating and quantification of IL-6^{+} **(D)** and TNF-α $^{+}$ fractions of treated B cells **(E)**. All used controls were set at 1 (dotted line). Data are shown as mean ± SEM. Paired t-tests were performed to compare groups. * p<0.05, ** p<0.01.

Supplementary Figure 5

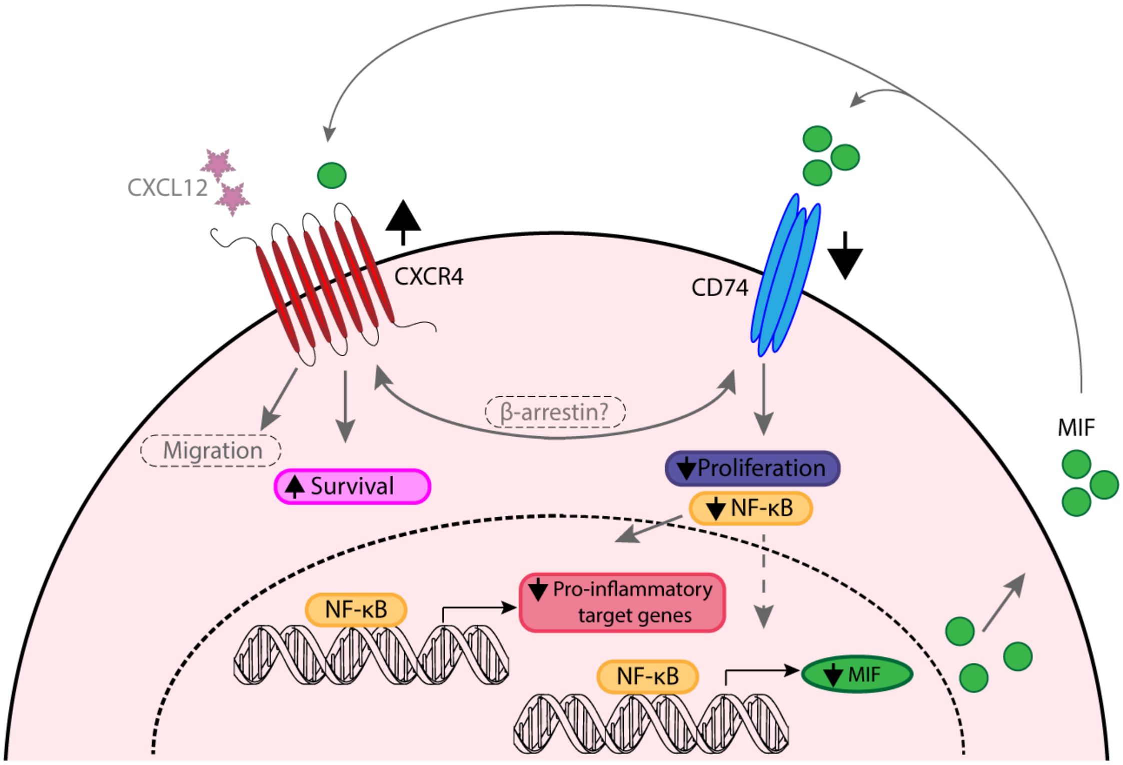

Supplementary Figure 5: Potential counter-regulatory mechanism and downstream effects of CD74 and CXCR4 signaling in peripheral B cells of early MS patients.

Our data demonstrate that CXCR4 is upregulated and CD74 is downregulated on peripheral B cells from early MS patients. Mechanistically, this counter-regulation of CXCR4 and CD74 could be explained by the higher affinity of MIF for CD74 than for CXCR4. After MIF-mediated internalization, CD74 potentially interacts with the adaptor molecule β-arrestin, preventing binding to and endocytosis of surface CXCR4. Functionally, peripheral B cells are less able to proliferate and express pro-inflammatory cytokines (MIF, IL-6, TNF-α) via NF-κB (mediated by CD74), but more capable of surviving peripheral tolerance checkpoints and infiltrating the central nervous system via local MIF production (mediated by CXCR4) in early MS. We postulate that within the central nervous system, $CXCR4^{hi}CD74^{lo}MIF^{lo}$ B cells are activated, proliferate and shift from a quiescent to a more pro-inflammatory state ($CXCR4^{lo}CD74^{hi}MIF^{hi}$).

Supplementary Table 1: Clinical information of patients and healthy controls

Cohorts		Patients, no	Gender, female no (%)	Age in years, median (IQR)[*]	Follow-up time in years, median (IQR)[*]	Disease duration in months, median (IQR) [†]
**FACS (B cells, ex vivo)**						
HC	Screening	15	12 (80%)	46 (32-60)	NA	NA
	Replication	20	16 (80%)	45 (32-60)	NA	NA
	Validation	20	13 (65%)	46 (24-60)	NA	NA
CIS	Total	33	24 (73%)	34 (22-48)	6.04 (2.17 - 9.81)	2.0 (0.1 - 4.0)
	Low-risk	17	12 (71%)	36 (22-48)	7.08 (5.21 - 9.81)	2.25 (1.0 - 4.0)
	High-risk	16	12 (81%)	32 (24-39)	4.08 (2.17 - 8.69)	2.0 (0.1 - 3.75)
RRMS	Screening	15	12 (80%)	46 (31-61)	NA	NA
	Replication	20	16 (80%)	46 (31-61)	NA	NA
	Validation	20	13 (65%)	46.5 (24-61)	NA	NA
**RT-PCR (B cells, ex vivo)**						
HC		22	14 (64%)	43.5 (24-66)	NA	NA
CIS	Total	18	13 (72%)	35.5 (24-48)	6.65 (2.17-9.81)	2.25 (1.0-4.0)
	Low-risk	13	10 (77%)	36 (25-48)	7.07 (5.21-9.81)	2.25 (1.0-4.0)
	High-risk	5	3 (60%)	32 (24-39)	3.36 (2.17-8.69)	3.0 (2.0-3.75)
RRMS		19	14 (74%)	42 (32-65)	NA	NA
**RT-PCR (B cells, in vitro)**						
HC		10	7 (70%)	30 (25-58)	NA	NA
CIS		9	7 (78%)	33 (23-39)	4.31 (1.34-10.99)	3 (1.5-5.25)
RRMS		9	8 (89%)	39 (21-54)	NA	NA
**ELISA (serum and plasma)**						
HC		29	19 (66%)	46 (24-60)	NA	NA
CIS	Total	61	43 (70%)	34,5 (19-51)	6.07 (2.17-9.81)	2.0 (0.0-4.0)
	Low-risk	33	24 (73%)	36 (22-51)	6.45 (3.78-9.81)	2.25 (0.5-4.0)
	High-risk	28	19 (68%)	33,5 (19-46)	5.51 (2.17-9.49)	2.0 (0.0-3.75)
RRMS		NA	NA	NA	NA	NA
**ELISA (culture medium)**						
HC		12	9 (75%)	40.8 (25-58)	NA	NA
CIS		12	8 (67%)	36 (23-47)	4.6 (0.55-8.53)	4.0 (0.0-6.0)
RRMS		12	9 (75%)	40.5 (21-66)	NA	NA

[*], at time of sampling; [†], time CIS to sampling; RRMS according to the McDonald 2010 criteria. Abbreviations: HC, healthy control; CIS, clinically isolated syndrome; IQR, interquartile range; NA, not applicable or available; RRMS, relapsing-remitting MS.

Supplementary Table 2: Monoclonal antibodies used for FACS

**Marker**	**Fluorochrome**	**Clone**	**Company**
CD3	AF700	SK7	Biolegend
CD14	APC	MOP9	BD Biosciences
CD19	BV785	HIB19	Biolegend
CD24	BV605	ML5	BD Biosciences
CD27	BV421	M-T271	BD Biosciences
CD38	PE-Cy7	HIT2	Biolegend
CD56	PE-CF594	B159	BD Biosciences
CD69	BV421	FN50	Biolegend
CD74	APC	LN2	Biolegend
CD95	BV605	DX2	Biolegend
CXCR4	PE-CF594	12G5	BD Biosciences
CXCR4	APC	12G5	BD Biosciences
HLA-DR	PerCP-Cy5.5	L243	Biolegend
IgA	FITC	IS11-8E10	Miltenyi-Biotec GmbH
IgD	PE-CF594	IA6-2	BD Biosciences
IgG	APC-H7	G18-145	BD Biosciences
IgM	BV510	MHM-88	Biolegend
IL-6	PE	MQ2-6A3	BD Biosciences
TNF	BV650	Mab11	BD Biosciences

Supplementary Table 3: Primer sequences used for real-time PCR

Gene	Forward primer	Reverse primer
MIF	ACCGCTCCTACAGCAAGC	CGCGTTCATGTCGTAATAGTTG
IL6	ATGAGTACAAAAGTCCTGATCCA	CTGCAGCCACTGGTTCTGT
TNF	GCCCAGGCAGTCAGATCATC	GGGTTTGCTACAACATGGGCT
NFKB1	CTGGCAGCTCTTCTCAAAGC	TCCAGGTCATAGAGAGGCTCA
