# Supplementary material for: The macrophage migration inhibitory factor pathway in human B cells is tightly controlled and dysregulated in multiple sclerosis
Source: Eur J Immunol. 2018 Sep 25;48(11):1861–71. doi: 10.1002/eji.201847623 (PMC6282801; doi:10.1002/eji.201847623)
Supplement: Supplementary file 1 — Supporting information [file EJI-48-1861-s001.pdf]

European Journal of Immunology

Supporting Information
for
DOI 10.1002/eji.201847623

Liza Rijvers, Marie-José Melief, Roos M. van der Vuurst de Vries, Maeva Stéphant, Jamie van Langelaar, Annet F. Wierenga-Wolf, Jeanet M. Hogervorst, Anneke J. Geurts-Moespot, Fred C. G. J. Sweep, Rogier Q. Hintzen and Marvin M. van Luijn

The macrophage migration inhibitory factor pathway in human B cells is tightly controlled and dysregulated in multiple sclerosis

© 2018 The Authors. *European Journal of Immunology* published by WILEY-VCH Verlag GmbH & Co. KGaA, Weinheim.

European Journal of Immunology

The macrophage migration inhibitory factor pathway in human B cells is tightly controlled and dysregulated in multiple sclerosis

Liza Rijvers, Marie-José Melief, Roos M. van der Vuurst de Vries, Maeva Stéphant, Jamie van Langelaar, Annet F. Wierenga-Wolf, Jeanet M. Hogervorst, Anneke J. Geurts-Moespot, Fred C. G. J. Sweep, Rogier Q. Hintzen and Marvin M. van Luijn

Correspondence: Dr. Marvin M. van Luijn, Postal address: Department of Immunology, Erasmus MC, University Medical Center, MS Center ErasMS, Wytemaweg 80, 3015 CN, Rotterdam, The Netherlands

---

Review Timeline:	Submission date:	28-Mar-2018
	First Editorial decision:	02-May-2018
	Revision/s received:	04-Jul-2018
	Accepted:	21-Aug-2018

Handling Executive Committee member: Prof. Jürgen Wienands

Please note that the correspondence below does not include the standard editorial instructions regarding preparation and submission of revised manuscripts, only the scientific revisions requested and addressed.

First Editorial Decision - 02-May-2018

Dear Dr. van Luijn,

Please accept our sincere apologies for the delay in the decision, there was a delay in receiving one of the referees' report.

Manuscript ID eji.201847623 entitled "The macrophage migration inhibitory factor pathway in human B cells: tight control and dysregulation in multiple sclerosis" which you submitted to the European Journal of Immunology has been reviewed. The comments of the referees are included at the bottom of this letter.

A revised version of your manuscript that takes into account the comments of the referees will be reconsidered for publication. Should you disagree with any of the referees' concerns, you should address

this in your point-by-point response and provide solid scientific reasons for why you will not make the requested changes.

You should also pay close attention to the editorial comments included below. **In particular, please edit your figure legends to follow Journal standards as outlined in the editorial comments. For all data, including the new data generated for the revision of the manuscript, please report in the Figure legends the number of independent experiments and number of samples per experiment (or experimental replicates). For flow cytometry data please show the full gating strategy, including the percentage of cells in the region or gate or event count. In the histograms/dot plots shown please report which fluorochromes were used and the scaling in the axis (log/lin). Failure to do this will result in delays in the re-review process.**

Please note that submitting a revision of your manuscript does not guarantee eventual acceptance, and that your revision will be re-reviewed by the referees before a decision is rendered.

If the revision of the paper is expected to take more than three months, please inform the editorial office. Revisions taking longer than six months may be assessed by new referees to ensure the relevance and timeliness of the data.

Once again, thank you for submitting your manuscript to European Journal of Immunology and we look forward to receiving your revision.

Yours sincerely,
Marta Vuerich

On behalf of
Prof. Jürgen Wienands

Dr. Marta Vuerich
Editorial Office
European Journal of Immunology
e-mail: ejied@wiley.com
www.eji-journal.eu

****

Reviewer: 1

Comments to the Author

This is an interesting study showing downregulation of the cytokine MIF in B cells of MS patients, correlating with a downregulation of the MIF-receptor CD74 and upregulation of the MIF receptor CXCR4. As this is not a pure correlation but there are also functional data involving anti-CD74 antibody and CXCR4 inhibitors influencing expression of the reciprocal other MIF-receptor or MIF knockdowns in a B cell line affecting CD74 expression, these data are convincing. The experiments are generally well performed.

What is missing is a better explanation of the data in the discussion. What is the model, how this counter-regulation works? Maybe the authors could present a model in a supplementary figure summarizing their data and suggesting how this regulation works.

Some more information should be provided:

-What is the affinity of MIF for CD74 as compared to CXCR4?

- CXCL12 is the main ligand for CXCR4, how does the affinity of these 2 cytokines (CXCL12 and MIF) for the same receptor compare?

- This counter-regulation of CXCR4 and CD74 on human B cells (as shown in Fig. 2D), has this also been observed in mouse cells? How about CD74 KO or CRCR4 KO, do they show effects on the other receptor?

-minor points: Fig.5C: the authors describe the effect of iso-1 as strong effects, however I would not call different expression levels of less than 50% strong effects.

-please clarify the effect of the anti-CD74 antibody. Does the treatment effect CD74 expression, is it agonistic, or antagonistic, i.e. blocking ligand binding?

-please write in the legend to Fig.6 how the B cells were activated.

Reviewer: 2

Comments to the Author

The Authors propose to study the MIF (macrophage migration inhibitory factor) pathway in human B cells by comparing healthy controls (HC) and multiple sclerosis patients.

Patients selection (with different categories: RRMS, CIS, high-risk CIS, CDM) and description is excellent and patients' numbers are adequate.

MIF is a ligand for CD74 and CXCR4. In murine B cells the interaction between CD74 and MIF results in enhanced proliferation and pro-inflammatory cytokine production. When MIF binds to CXCR4 induces cell migration.

In this paper the expression of CXCR4 and CD74 was studied by flow cytometry. The data shows that, in

RRMS, the expression of CXCR4 is increased and that of CD74 is reduced, resulting in a higher CXCR4/CD74 ratio. The comparison between low-risk and high-risk CIS shows that a high CXCR4/CD74 ratio indicates a more severe disease. In the second part of the paper, after a series of in vitro experiments, the Authors conclude that, in human, CD74 has a pro-inflammatory effect whereas CXCR4 makes cells less sensitive to FAS-mediated apoptosis.

The Authors do not discuss how these functions influence MS evolution.

The patients and data are interesting, but the paper is confused.

1. If we consider the data presented in Fig1 and 2 together (below, I plotted the numbers reported in Fig1 and 2 together), we observe that the CXCR4/CD74 ratio in CIS is lower than in HD. This is caused by the increased expression of CD74 in CIS.

Fig. 3 The inverse expression of CXCR4 and CD74 in B cell development is clearly demonstrated. The comparison between RRMS and high-risk CIS show that CXCR4 is more expressed and CD74 less expressed in every B cell population in RRMS (see graphs scales). This is not mentioned or discussed. It would be interesting to know whether the different B cell populations are equally represented in HC and patients (more memory B cells may result in more CD74 and less CXCR4).

The sentence (page 6) "These data demonstrate that MIF receptors CXCR4 and CD74 are inversely expressed on B cells, which is dysregulated during early disease onset in MS" is wrong. The regulated expression during development is maintained in patients. In early onset MS each population of B cells expresses more CD74 (IgG memory B cells have a MIF around 2700 in CIS and less than 1500 in RRMS).

Fig.4 MIF in the serum is similar in HC and MS, but MIF mRNA is lower in CIS and a high ratio is associated to low MIF mRNA.

Fig.5 inhibition of MIF signaling increases CXCR4 and reduces CD74 expression. Anti CD74 increases CXCR4 and reduces MIF. Inhibition of CXCR4 increases MIF.

Fig.6 OK

In the discussion line 29-30 selection of transitional into naïve B cells does not occur in the germinal centers.

The hypothesis that MIF in the brain may attract B cells of MS patients through CXCR4 is attractive. It is not clear to me how locally high MIF would trigger endogenous MIF production and inflammation (role of CD74? But how?).

Reviewer: 3

Comments to the Author

Report on EJIMM- eji.201847623

European Journal of Immunology

Rijvers and colleagues studied the role of the pleiotropic, chemokine-like inflammatory cytokine MIF in multiple sclerosis (MS). The particular focus of this study was on the role of the MIF receptors CD74 and CXCR4 in B cells and their link to MS pathology. They wanted to explore the mechanisms underlying the survival of MS B cell and their survival of peripheral tolerance checkpoints to mediate local inflammation. MIF pathways had previously been described to control B-cell development in physiology and diseases such as experimental autoimmune encephalomyelitis (EAE), B-CLL, and atherosclerosis. Rijvers et al. found that MIF and the MIF receptor CD74 are downregulated in B cells from early onset MS patients, while the MIF receptor CXCR4 was observed to be upregulated. They also suggest that B cells are the main blood population expressing MIF. The reciprocal expression profile of the MIF receptors CD74 and CXCR4 on B cells appears to be functionally linked, as blocking of MIF and CD74 signaling triggered CXCR4 expression and vice versa. Although the study does not really deeply explore the mechanisms behind this reciprocal relationship, they found that it is associated distinct effects on pro-inflammatory activity, proliferation and sensitivity of B cells to apoptosis. The highlight of the study is the identified reciprocal negative regulation loop between CD74 and CXCR4 in human B cells in early onset MS that has not previously been noted. It could help to understand how pathogenic B cells survive peripheral tolerance checkpoints to mediate disease activity in early MS.

Criticism and questions

1. Is there a reason why the two RRMS cohorts (n=15, Figure 1; n=20, Supplementary Figure 1B) were analyzed separately rather than as one-joint RRMS cohort?

a. Authors write in the legend of Supplementary Figure 1: “Similar experiments were performed for B cells from an additional cohort of RRMS patients (n=20) and matched HC (n=20, see Supplementary Experimental Procedures; B).”

2. It would be better to perform simultaneous stainings of CXCR4high /CD47low cells and populations and to calculate the ratios from that analysis. This would give even more (reciprocal) receptor expression data.

3. The title could be more specific; it reads a bit like a title of a review article.

4. The functional assays are not fully convincing and do not really fit to the early onset MS phenotype. One reason is that the experiments were done WITHOUT addition of exogenous MIF, so apparently solely relying on “autocrine” MIF loops??

5. In Figure 6, NFkB and TNF should be analyzed on protein level (i.e. p-p65, Ikba, secreted TNF). This would be more meaningful than the mRNA levels.

6. Recent literature on MIF and B cells as well as autoantigens in atherosclerosis was ignored or forgotten.

European Journal of Immunology

First Revision – authors' response 04-Jul-2018

Comments Reviewer 1: This is an interesting study showing downregulation of the cytokine MIF in B cells of MS patients, correlating with a downregulation of the MIF-receptor CD74 and upregulation of the MIF receptor CXCR4. As this is not a pure correlation but there are also functional data involving anti-CD74 antibody and CXCR4 inhibitors influencing expression of the reciprocal other MIF-receptor or MIF knockdowns in a B cell line affecting CD74 expression, these data are convincing. The experiments are generally well performed.
What is missing is a better explanation of the data in the discussion. What is the model, how this counter-regulation works? Maybe the authors could present a model in a supplementary figure summarizing their data and suggesting how this regulation works.
Reply: Thank you for this comment. In the Discussion section, we do indicate how CD74 downregulation (second paragraph, p8-9) and CXCR4 upregulation (third paragraph, p9) on B cells potentially link to developmental and functional changes in the context of autoimmunity. It is very likely that the observed CD74loCXCR4hi phenotype of B cells associates with the enhanced ability of autoreactive, naive B-cell populations to escape from peripheral tolerance checkpoints in MS (see ref 3 of the manuscript). Currently, far less is clear about the counter-regulatory mechanism of CD74 and CXCR4. One explanation for the low CD74 and high CXCR4 expression on B cells in MS could be that MIF-dependent endocytosis of CD74 contributes to the interaction with adaptor molecule β-arrestin, preventing binding to and internalization of surface CXCR4 (Xie, PLoS One 2011; Orsini, J Biol Chem 1999; van der Vorst, J Mol Med (Berl) 2015). We now included this interpretation in the text at p9-10, as well as a graphical model summarizing our data and demonstrating possible mechanisms of action (new Supplementary Figure 5).

Some more information should be provided: -What is the affinity of MIF for CD74 as compared to CXCR4?
Reply: The affinity of MIF for CD74 is 9 nM (Leng, J Exp Med 2003), while that for CXCR4 is 19 nM (Bernhagen, Nat Med 2007). This supports our new interpretation that MIF preferentially binds to CD74, resulting in its uptake and potential binding to β-arrestin (see above). Due to this counter-regulation of CD74 and CXCR4 in B cells, however, MIF may become more available for CXCR4, contributing to their insensitivity to Fas-mediated apoptosis (shown in this paper) and entry into the CNS (refs 36, 37) in MS patients. This information is now added and discussed in the Discussion section (p9-10).

- CXCL12 is the main ligand for CXCR4, how does the affinity of these 2 cytokines (CXCL12 and MIF) for the same receptor compare?
Reply: The affinity of CXCL12 for CXCR4 is 3.6 nM, so this is higher than MIF (19 nM; see also Crump, EMBO J 1997). CXCL12 can thus interfere with the binding of MIF to CXCR4, but this will largely depend

on the local availability of these two cytokines. Although CXCL12 was shown to be enriched in the CNS of MS patients, this did not coincide with local B cell infiltration (see p10 in Discussion). So, although not the scope of the paper, probably MIF (elevated in MS brain, ref 36 of the paper) attracts CXCR4hi B cells to the CNS, where CXCL12 is most likely contributes to the follicle-like B cell organization as found in MS brain (Allen, Immunity, 2007; Serafini, Brain Pathol, 2004; McCandless, Am J Pathol, 2008), resulting in local reactivation. Functional differences between MIF and CXCL12 binding to CXCR4 were also previously described (Rajesekaran, J Biol Chem 2016) and deserves further investigation

2- This counter-regulation of CXCR4 and CD74 on human B cells (as shown in Fig. 2D), has this also been observed in mouse cells? How about CD74 KO or CRCR4 KO, do they show effects on the other receptor?

Reply: To our knowledge, no studies have been performed in (B cell-restricted) CXCR4 or CD74 KO mice with regard to the co-regulation of these receptors in B cells. CXCR4 KO mice die prenatally and have defective nervous and hematopoietic systems, including strongly reduced B-lymphopoiesis (Ma, PNAS 1998). For CD74 KO mice, impaired B-cell development and function was found (refs 20, 21 of the paper), but the effects on CXCR4 expression were not addressed. Although conclusive evidence is missing, CXCR4 downstream molecule ZAP-70 did seem to be more phosphorylated in CD74-deficient murine B cells after CXCL12 stimulation (Klasen, J Immunol 2014). Additionally, in a study by Frölich and colleagues, human B cells showed increased migration towards CXCL12 after treatment with milatuzumab, a specific humanized antibody against CD74 (Arthritis Res Ther 2012). These findings support the positive effects of CD74 inhibition on CXCR4 expression by B cells as seen in our study. This is now discussed and abovementioned references were added to the manuscript (p9-10).

-minor points:

Fig.5C: the authors describe the effect of iso-1 as strong effects, however I would not call different expression levels of less than 50% strong effects.

Reply: We agree with the reviewer and removed ‘strong’ from the text accordingly (p6).

-please clarify the effect of the anti-CD74 antibody. Does the treatment effect CD74 expression, is it agonistic, or antagonistic, i.e. blocking ligand binding?

Reply: LN2 is a neutralizing anti-human CD74 antibody (Klasen, J Immunol 2014; Leng, J Exp Med 2003). We added this information to the text in the Materials and Methods (p13).

-please write in the legend to Fig.6 how the B cells were activated.

Reply: We now indicated in this legend that B cells were activated with anti-IgM (p20).

Comments Reviewer 2:

The Authors propose to study the MIF (macrophage migration inhibitory factor) pathway in human B cells by comparing healthy controls (HC) and multiple sclerosis patients. Patients selection (with different categories: RRMS, CIS, high-risk CIS, CDM) and description is excellent and patients' numbers are adequate. MIF is a ligand for CD74 and CXCR4. In murine B cells the interaction between CD74 and MIF results in enhanced proliferation and pro-inflammatory cytokine production. When MIF binds to CXCR4 induces cell migration. In this paper the expression of CXCR4 and CD74 was studied by flow cytometry. The data shows that, in RRMS, the expression of CXCR4 is increased and that of CD74 is reduced, resulting in a higher CXCR4/CD74 ratio. The comparison between low-risk and high-risk CIS shows that a high CXCR4/CD74 ratio indicates a more severe disease. In the second part of the paper, after a series of in vitro experiments, the Authors conclude that, in human, CD74 has a pro-inflammatory effect whereas CXCR4 makes cells less sensitive to FAS-mediated apoptosis. The Authors do not discuss how these functions influence MS evolution.

The patients and data are interesting, but the paper is confused. 1. If we consider the data presented in Fig1 and 2 together (below, I plotted the numbers reported in Fig1 and 2 together), we observe that the CXCR4/CD74 ratio in CIS is lower than in HD. This is caused by the increased expression of CD74 in CIS.

Reply: B cells were analyzed for CXCR4 and CD74 expression differences in two independent studies (HD vs RRMS, Fig. 1 and CIS-CIS vs CIS-CDMS, Fig. 2). Per study, different antibody panels, antibody batches and flow cytometer settings were used. For these reasons, the MFI of these markers cannot be compared between HD and CIS.

Fig. 3 The inverse expression of CXCR4 and CD74 in B cell development is clearly demonstrated. The comparison between RRMS and high-risk CIS show that CXCR4 is more expressed and CD74 less expressed in every B cell population in RRMS (see graphs scales). This is not mentioned or discussed.

Reply: Please see our comment above.

It would be interesting to know whether the different B cell populations are equally represented in HC and patients (more memory B cells may result in more CD74 and less CXCR4).

Reply: We thank the reviewer for raising this valid point. In our screening cohort (Fig. 1, Supplementary Table 1), naive B-cell frequencies were higher in RRMS versus HD blood. However, we were unable to validate this in our additional cohort (Supplementary Fig. 1; Supplementary Table 1). Also no differences in frequencies were found between the CIS-CIS and CIS-CDMS groups. Nevertheless, the differences in CXCR4 and CD74 expression on B cells were consistent for all three early MS groups compared to the corresponding control groups. Additional analysis reveals that also on B-cell subset level, CXCR4/CD74 expression ratios are significantly higher for RRMS patients as compared to HD from the same screening cohort (Supplementary Table 1 and new Supplementary Fig. 2)

The sentence (page 6) “These data demonstrate that MIF receptors CXCR4 and CD74 are inversely expressed on B cells, which is dysregulated during early disease onset in MS” is wrong. The regulated expression during development is maintained in patients. In early onset MS each population of B cells expresses more CD74 (IgG memory B cells have a MIF around 2700 in CIS and less than 1500 in RRMS).

Reply: To our view, the differences found between RRMS and HD as well as CIS-CIS and CIS-CDMS groups is ample evidence to state that the inverse expression of CXCR4 and CD74 on B cells is dysregulated during early MS onset. As also commented above, the different use of antibody panels, antibody batches and flow cytometer settings in the two independent studies makes it impossible to compare CD74 and CXCR4 MFI data between CIS and RRMS groups. Although not the scope of the study, another independent FACS screen using the same panels and settings as well as matching samples for age and gender should be performed to claim that CD74 (or CXCR4) is differently expressed on B cells between these groups.

Fig.4 MIF in the serum is similar in HC and MS, but MIF mRNA is lower in CIS and a high ratio is associated to low MIF mRNA.

Reply: We changed the title of this figure legend into: ‘Serum MIF levels are not different, while the predominant MIF expression in B cells is reduced in CIS and RRMS patients’.

Fig.5 inhibition of MIF signaling increases CXCR4 and reduces CD74 expression. Anti CD74 increases CXCR4 and reduces MIF. Inhibition of CXCR4 increases MIF.

Reply: We changed the title of this figure legend into: ‘Interference with MIF signaling pathways reveal mutual regulation of CD74, CXCR4 and MIF expression in in vitro-activated B cells’.

Fig.6 OK

In the discussion line 29-30 selection of transitional into naïve B cells does not occur in the germinal centers.

Reply: We agree with the reviewer and changed ‘germinal centers’ into ‘secondary lymphoid organs’.

The hypothesis that MIF in the brain may attract B cells of MS patients through CXCR4 is attractive. It is not clear to me how locally high MIF would trigger endogenous MIF production and inflammation (role of CD74? But how?).

Reply: Based on our data, we postulate that after their attraction into the CNS by exogenous MIF, MIFloCD74loCXCR4hi (peripherally quiescent) B cells are locally re-activated by CD4+ Th cells, resulting in their enhanced expression and secretion of endogenous MIF (see also Fig. 5A, B). This local activation of B cells will then cause CD74 upregulation and CXCR4 downregulation (unpublished data and Fig. 3), enhancing their recognition of MIF via CD74 (higher affinity than CXCR4; see comment reviewer 1). Consequently, both the pro-inflammatory and proliferative capacity of these B cells increases (see Fig. 6) to mediate CNS inflammation. We now clarified this implication in the Discussion (p9-10).

Comments Reviewer 3: Rijvers and colleagues studied the role of the pleiotropic, chemokine-like inflammatory cytokine MIF in multiple sclerosis (MS). The particular focus of this study was on the role of the MIF receptors CD74 and CXCR4 in B cells and their link to MS pathology. They wanted to explore the mechanisms underlying the survival of MS B cell and their survival of peripheral tolerance checkpoints to mediate local inflammation. MIF pathways had previously been described to control B-cell development in physiology and diseases such as experimental autoimmune encephalomyelitis (EAE), B-CLL, and atherosclerosis. Rijvers et al. found that MIF and the MIF receptor CD74 are downregulated in B cells from early onset MS patients, while the MIF receptor CXCR4 was observed to be upregulated. They also suggest that B cells are the main blood population expressing MIF. The reciprocal expression profile of the MIF receptors CD74 and CXCR4 on B cells appears to be functionally linked, as blocking of MIF and CD74 signaling triggered CXCR4 expression and vice versa. Although the study does not really deeply explore the mechanisms behind this reciprocal relationship, they found that it is associated distinct effects on pro-inflammatory activity, proliferation and sensitivity of B cells to apoptosis. The highlight of the study is the identified reciprocal negative regulation loop between CD74 and CXCR4 in human B cells in early onset MS that has not previously been noted. It could help to understand how pathogenic B cells survive peripheral tolerance checkpoints to mediate disease activity in early MS. Criticism and questions 1. Is there a reason why the two RRMS cohorts (n=15, Figure 1; n=20, Supplementary Figure 1B) were analyzed separately rather than as one-joint RRMS cohort?

Reply: Yes. We first analyzed CXCR4 and CD74 expression differences on B cells from a screening cohort of RRMS patients and HC. Significant differences were then confirmed in a second RRMS and HC cohort. Because of the use of different antibody panels, antibody batches and flow cytometer settings in these two independent studies, MIF data could not be pooled and RRMS cohorts had to be analyzed separately.

a. Authors write in the legend of Supplementary Figure 1: “Similar experiments were performed for B cells from an additional cohort of RRMS patients (n=20) and matched HC (n=20, see Supplementary Experimental Procedures; B).

Reply: We apologize for this textual error in the legend and changed ‘Supplementary Experimental Procedures’ into ‘Supplementary Table 1’

2. It would be better to perform simultaneous stainings of CXCR4high /CD47low cells and populations and to calculate the ratios from that analysis. This would give even more (reciprocal) receptor expression data.

Reply: We actually performed simultaneous staining for CXCR4 and CD74, so the antibodies were in the same FACS tube and ratios were calculated for the same cells. To exemplify the reciprocal expression of CXCR4 and CD74 on B cells, a representative FACS plot as well as the quantification of the % CXCR4hiCD74lo B cells was included as Supplementary Fig. 1C and 1D.

3. The title could be more specific; it reads a bit like a title of a review article.
Reply: We agree and adapted the title into: 'The macrophage migration inhibitory factor pathway in human B cells is tightly controlled and dysregulated in multiple sclerosis'

4. The functional assays are not fully convincing and do not really fit to the early onset MS phenotype. One reason is that the experiments were done WITHOUT addition of exogenous MIF, so apparently solely relying on "autocrine" MIF loops??
Reply: Our in vitro experiments in Fig. 5 demonstrate that inhibition of MIF resulted in CD74 downregulation and CXCR4 upregulation on activated B cells. CXCR4 inhibition resulted in MIF and CD74 upregulation, whereas anti-CD74 treatment downregulated MIF and upregulated CXCR4. To our view, this reciprocal regulation of MIF, CD74 and CXCR4 expression does support the CXCR4hiCD74loMIFlo phenotype observed for ex vivo B cells in early MS patients. We do agree that the use of exogenous MIF would have helped in confirming our results. However, after testing several batches of commercially available MIF (R&D Systems, Biolegend) and homemade MIF (kind gift from Prof. Calandra and Dr. Roger, Lausanne), we were not able to detect any human B-cell activation based on qPCR, FACS and calcium flux assays. It is generally recognized that the use of biologically active MIF (having a very short half-life) for functional assays with primary human cells is extremely difficult. This is probably also the reason why hardly any studies have been published using rhMIF in this context.

5. In Figure 6, NFkB and TNF should be analyzed on protein level (i.e. p-p65, Ikba, secreted TNF). This would be more meaningful than the mRNA levels.
Reply: We now performed additional experiments using B cells that were activated by anti-IgM with and without the addition of anti-CD74 antibody (LN2) or CXCR4 antagonist (AMD3100) for 1 and 3 days. NF-κB1 protein p105 (the protein that is directly translated from the mRNA analyzed in Fig. 6) was less expressed under CD74 blocking conditions, as determined by Western Blotting after 24h. Furthermore, 72h after stimulation, cells were collected and analyzed for intracellular IL-6 and TNF-α production using FACS. IL-6 and TNF-a protein expression was strongly impaired after blocking with LN2 and not with the use of AMD3100. These results support our qPCR data (Fig. 6) and are now mentioned in the manuscript text (p7) and added as Supplementary Fig. 4.

6. Recent literature on MIF and B cells as well as autoantigens in atherosclerosis was ignored or forgotten.
Reply: The recent publication by Schmitz and colleagues (FASEB J 2018) indeed nicely corresponds with our current findings on MIF regulation, B cells and MS. We now added this study on this important matter as a reference to the Introduction (p3) and Discussion (p9).

Second Editorial Decision - 07-Aug-2018

Dear Dr. van Luijn,

It is a pleasure to provisionally accept your manuscript entitled "The macrophage migration inhibitory factor pathway in human B cells is tightly controlled and dysregulated in multiple sclerosis" for publication in the European Journal of Immunology. For final acceptance, please follow the instructions below and return the requested items as soon as possible as we cannot process your manuscript further until all items listed below are dealt with.

Please note that EJI articles are now published online a few days after final acceptance (see Accepted Articles: https://onlinelibrary.wiley.com/toc/15214141/0/ja). The files used for the Accepted Articles are the final files and information supplied by you in Manuscript Central. You should therefore check that all the information (including author names) is correct as changes will NOT be permitted until the proofs stage.

We look forward to hearing from you and thank you for submitting your manuscript to the European Journal of Immunology.

Yours sincerely,
Laura Soto Vazquez

on behalf of
Prof. Jürgen Wienands

Editorial Office
European Journal of Immunology
e-mail: ejied@wiley.com
www.eji-journal.eu
